# Fluorination of Naturally Occurring *N*^6^-Benzyladenosine Remarkably Increased Its Antiviral Activity and Selectivity

**DOI:** 10.3390/molecules22071219

**Published:** 2017-07-20

**Authors:** Vladimir E. Oslovsky, Mikhail S. Drenichev, Liang Sun, Nikolay N. Kurochkin, Vladislav E. Kunetsky, Carmen Mirabelli, Johan Neyts, Pieter Leyssen, Sergey N. Mikhailov

**Affiliations:** 1 Engelhardt Institute of Molecular Biology, Russian Academy of Sciences, Moscow 119991, Russia; vladimiroslovsky@gmail.com (V.E.O.); mdrenichev@mail.ru (M.S.D.); nikola.76@mail.ru (N.N.K.); kunvladea@gmail.com (V.E.K.); 2 Laboratory for Virology and Chemotherapy, Department of Microbiology and Immunology, Rega Institute for Medical Research, KU Leuven-University of Leuven, Minderbroedersstraat 10, Leuven 3000, Belgium; liang.sun@kuleuven.be (L.S.); carmen.mirabelli@kuleuven.be (C.M.); johan.neyts@kuleuven.be (J.N.); pieter.leyssen@kuleuven.be (P.L.)

**Keywords:** fluorinated *N*^6^-benzyladenosines, synthesis and antiviral activity, SAR, enterovirus 71

## Abstract

Recently, we demonstrated that the natural cytokinin nucleosides *N*^6^-isopentenyladenosine (**iPR**) and *N*^6^-benzyladenosine (**BAPR**) exert a potent and selective antiviral effect on the replication of human enterovirus 71. In order to further characterize the antiviral profile of this class of compounds, we generated a series of fluorinated derivatives of **BAPR** and evaluated their activity on the replication of human enterovirus 71 in a cytopathic effect (CPE) reduction assay. The monofluorination of the **BAPR**-phenyl group changed the selectivity index (SI) slightly because of the concomitant high cell toxicity. Interestingly, the incorporation of a second fluorine atom resulted in a dramatic improvement of selectivity. Moreover, *N*^6^-trifluoromethylbenzyladenosine derivatives (**9**–**11**) exhibited also a very interesting profile, with low cytotoxicity observed. In particular, the analogue *N*^6^-(3-trifluoromethylbenzyl)-adenosine (**10**) with a four-fold gain in potency as compared to **BAPR** and the best SI in the class represents a promising candidate for further development.

## 1. Introduction

For many years, natural products (NPs) have been a leading source for the majority of the approved drugs, and their structures are a valuable source of inspiration for medicinal chemists [1]. Interestingly, only 36% of the new chemical entities discovered between 1981 and 2010 were developed without inspiration from a natural product [2].

Among natural products, the development of nucleosides is by far the most fruitful field of investigation. About one hundred drugs derive from nucleoside structures: the vast majority of them were developed as antiviral drugs, and a consistent proportion as antitumor drugs. Natural nucleosides are isolated from DNA, RNA, nucleotides, and coenzymes of various natural sources. Nowadays, the nucleoside library consists of about 550 compounds, and is a promising pool for the development of new biologically active compounds [3,4,5].

*N*^6^-Modified purine nucleosides (cytokinin nucleosides) are an important group of biologically active natural compounds with a unique spectrum of biological activities [3]. Cytokinin nucleosides contain a hydrophilic ribofuranose moiety and a purine heterocyclic scaffold modified with a hydrophobic residue at the *N*^6^ position. tRNA contains *N*^6^-isopentenyladenosine and some related nucleosides [6,7]. *N*^6^-Substituted adenosines are naturally present in plants [8,9,10] and bacteria [11].

In 2008, Arita and co-workers found that *N*^6^-benzyladenosine (**BAPR**) exhibited a pronounced antiviral activity against the replication of human enterovirus 71 (EV71) [12]. EV71 is a non-enveloped, single-stranded, positive-sense RNA virus belonging to the *Enterovirus* genus within the *Picornaviridae* family. EV71 commonly causes hand-, foot-, and mouth disease (HFMD), a mild and self-limiting illness mostly affecting children under the age of five. In some patients, EV71 has been associated with severe neurological complications including encephalitis, aseptic meningitis, and acute flaccid paralysis [13,14,15]. EV71 is prevalent worldwide, but most of the large outbreaks of neurotropic EV71 have occurred in the Pacific-Asia area [15,16,17]. However, in recent years, such epidemic tracts have been reported also in America and in Europe [15,17]. The World Health Organization has placed EV71 as one of the next biggest worldwide threats to public health, especially to young children, due to the lack of effective antiviral treatments [18,19].

Recently, we showed that, similarly to **BAPR**, two other naturally occurring plant cytokinin nucleosides, namely *N*^6^-isopentenyladenosine and *N*^6^-furfuryladenosine (kinetin riboside), possessed a potent and selective antiviral effect on EV71 [20,21]. Unfortunately, these compounds were rather cytotoxic, with CC_50_ values in the low micromolar range (4–8 μM). We were able to improve the selectivity of this group of compounds by modifying the size and the nature of the linker. In particular, a modified **BAPR** with a two-to-three atom-long linker had a very pronounced antiviral activity, and a 50-fold improvement of the selectivity index (SI) as result of a lower cytotoxicity [21].

**Figure 1:**
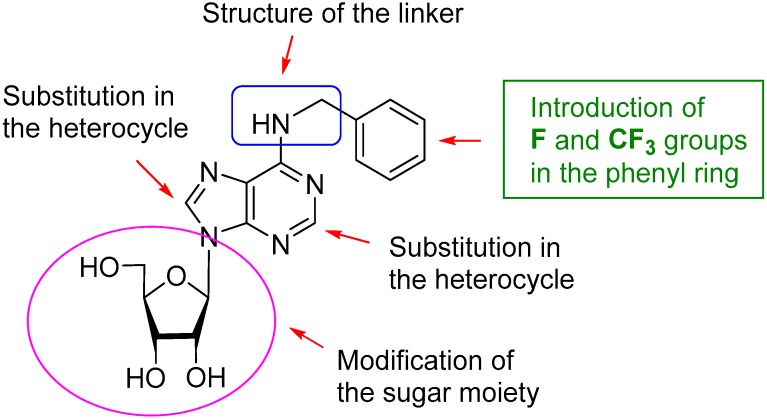
Strategy of modification of natural cytokinin nucleoside BAPR.

The introduction of fluorine in order to improve the pharmacological properties of a drug is a modern trend in medicinal chemistry. Currently, there are about 200 fluorinated drugs on the market (~20% of all pharmaceuticals), with even higher figures for agrochemicals (up to 30%) [22,23]. Therefore, in the present study, we report on the modification of natural **BAPR** by the substitution in the phenyl ring with fluoro-, difluoro-, and trifluorometyl groups to evaluate the eventual improvement in the antiviral profile of these fluorinated compounds in the context of EV71 replication (Figure 1).

## 2. Results and Discussion

### 2.1. Chemistry

Recently, we have developed a new useful and versatile approach for the preparation of *N*^6^-modified adenosine derivatives by the regioselective *N*^6^-alkylation of *N*^6^-acetyl-2′,3′,5′-tri-*O*-acetyladenosine with alcohols under Mitsunobu reaction conditions or with alkyl halides promoted by a base [20,21,24,25]. The main advantage of our method is the possibility to use both alkyl halides and alcohols for *N*^6^-modification. This is important, especially in the case when an amine is not stable or is hardly available. Using this methodology, several hundred *N*^6^-substituted adenosines have been synthesized in one of our laboratories.

The traditional approach for the preparation of *N*^6^-alkylated or *N*^6^-arylated adenosines is the substitution of the chlorine atom in commercially available 6-Chloropurine riboside with alkyl- or arylamines [26,27]. 6-hloropurine riboside can be readily prepared by the deacetylation of 2′,3′,5′-tri-*O*-acetyl-6-chloropurine riboside [28].

To simplify the separation procedure, we used 2′,3′,5′-tri-*O*-acetyl-6-chloropurine riboside directly in the substitution reactions. The acetyl groups are completely preserved in the reaction with aniline, and the protected intermediate can be isolated by silica gel chromatography and characterized. After the removal of the acetyl groups by ammonolysis, *N*^6^-phenyladenosine was obtained in overall high yield [21]. On the other hand, the reaction of 2′,3′,5′-tri-*O*-acetyl-6-chloropurine riboside with benzylamines was accompanied by the formation of by-products due to the partial removal of the protective groups, which complicated the chromatographic control of the reaction, and required a large excess of amines for the full conversion of the starting compound. Therefore, we decided to study the stability of different *O*-acyl groups to select the one optimal for our purposes. The results of the *O*-deacylation experiments of 2′,3′,5′-tri-*O*-acylinosine are summarized in Table 1.

**Scheme 1:**
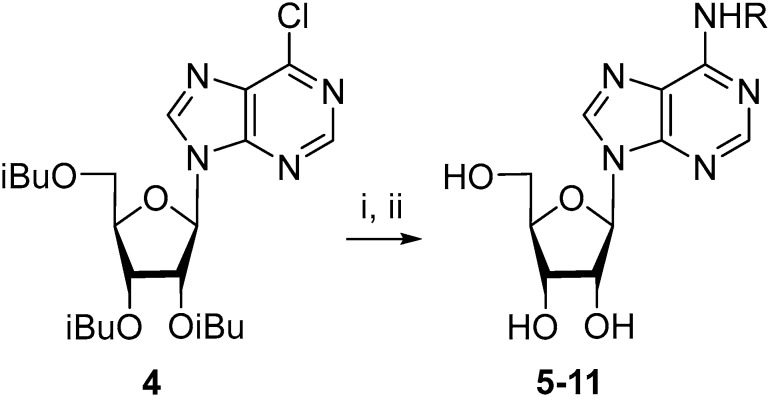
Synthesis of *N*^6^-alkyladenosines by the substitution of the chlorine atom in 2′,3′,5′-tri-*O*-isobutyroyl-6-chloropurineriboside. Reagents and conditions: (i) RNH_2_, DIPEA, MeCN, 70 °C, 10–24 h; (ii) MeNH_2_/EtOH, room temperature., 24 h, 50–98% (overall yields); (The structure of R is given in Table 1).

According to the data in Table 1, the acetyl group is rather labile under basic conditions, and the benzoyl group is the most stable. The properties of the iso-butyroyl group exhibited the best behavior for our aims, since it is fairly resistant to the action of benzylamines, and its synthesis is more straightforward than that of the benzoyl derivatives. The compound 2′,3′,5′-tri-*O*-isobutyroyl-6-chloropurine riboside (**4**) has then been used as the starting substrate in the reactions with a small excess of benzylamines with fluoro- and trifluoromethyl groups (Scheme 1). The protective groups were removed in the presence of MeNH_2_/EtOH at room temperature with the subsequent chromatographic purification of the resulting products. Compounds **5**–**11** were obtained in overall good yield (50–98%). It should be mentioned that some of these compounds were previously prepared starting from 6-chloropurine riboside [26].

The structure of the obtained compounds was confirmed by NMR and mass spectroscopy. The presence of fluorine atoms in the phenyl residue was confirmed by spin-spin coupling constants between ^19^F and ^1^H in ^1^H-NMR spectra (*J*_H-F_) and between ^19^F and ^13^C in ^13^C-NMR spectra (*J*_C-F_). The ^1^H-NMR spectra of the fluorinated *N*^6^-benzyladenosine analogues (**5**–**8**) in the low field region were complicated by the presence of ^19^F-^1^H couplings: ^3^*J*_H-F_–8.0–9.0 Hz, ^4^*J*_H-F_–6.7–5.5 Hz, and ^5^*J*_H-F_ ˂ 2.0 Hz. In the ^13^C-NMR spectra, three types of coupling constants *J*_C-F_ were present, which are characteristic of fluorinated aromatic compounds [29]: ^1^*J*_C-F_–240–248 Hz, ^2^*J*_C-F_–12–24 Hz, and ^3^*J*_C-F_–7.5 Hz. The presence of trifluoromethyl residue in nucleosidic derivatives (**9**–**11**) was confirmed by low-intensive quartet with a coupling constant of ~30 Hz in ^13^C-NMR spectra. This constant was consistent with the literature data for trifluoromethylated aromatic compounds [29]. Despite the majority of the synthesized compounds having been characterized by NMR earlier, their detailed analysis and the assignment of all chemical shifts and coupling constants has not been presented. Therefore, we provided in the Supplementary section a detailed NMR analysis for each analogue produced.

### 2.2. Biological Activity on EV71 and Structure-Activity Relationship (SAR)

We have shown earlier that three natural cytokinin nucleosides (compound **1**–**3**) exerted a potent antiviral effect on the replication of EV71 with an EC_50_ of 0.3–1.4 μM, but exhibited also a rather high cytotoxicity [20,21] (Table 2). As previously mentioned, modifications of the *N*^6^-substituent (linker) of the **BAPR** scaffold led to a remarkable increase of selectivity [21]. Here, we produced a series of **BAPR** analogues to evaluate the effect of the fluorination of **BAPR** on the replication of EV71. A cytopathic effect (CPE) reduction assay was performed with the newly synthetized analogues (compounds **5**–**11**) in rhabdomyosarcoma (RD) cells. **BAPR**, *N*^6^-isopenthenyladenosine, and *N*^6^-furfuryladenosine were included in the screening, and the toxicity of all of the aforementioned compounds was evaluated in parallel on treated-uninfected cells.

Overall, the incorporation of fluoro- and trifluoromethyl groups significantly improved the selectivity index of **BAPR** (Table 2). In particular, the monofluorination of the phenyl group (compounds **5**–**7**) slightly changed the SI because of the concomitant cytotoxicity of such compounds. Surprisingly, the incorporation of a second fluorine atom resulted in a substantial improvement over the selectivity. In particular, compound **8** presented an EC_50_ comparable to **BAPR** with a dramatic reduction of cell toxcity: from a CC_50_ of 13.3 ± 3.7 μM for the monofluorinated analogue to a CC_50_ higher than 250 μM for the difluorinate counterpart. We wanted also to assess the effect of a trifluoromethyl group on the phenyl ring of **BAPR** on EV71 replication. Compounds **9**, **10** and **11** did not show any cytotoxicity at the highest concentration tested, and the analogue *N*^6^-(3-trifluoromethylbenzyl)-adenosine (compound **10**) exhibited also a four-fold improvement in potency as compared to **BAPR**.

Previous reports showed that the halogenation (and, in particular, the addition of I or Cl atoms) on a BAPR scaffold increased its selectivity by reducing the cell toxicity in cancer cell lines [26,30]. In line with these findings, we observed that the gain in selectivity in our model was mostly due to a decreased cell toxicity. In particular, only the analogues containing two fluorine or a trifluoromethyl group dramatically improved the cytotoxicity. In spite of our interest in understanding compound-driven cell toxicity, addressing this question was beyond our scientific scope. Future works on the optimization of this class of analogues may shed light on the mechanism of action and their metabolization within an infected cell.

Altogether, our data revealed that the introduction of at least two fluorine atoms or a trifluoromethyl group on the phenyl ring of **BAPR** dramatically improved its selectivity by reducing the cytotoxicity, and in case of compound **10**, also by increasing the potency.

## 3. Materials and Methods

### 3.1. General

The solvents and materials were reagent grade and were used without additional purification. Column chromatography was performed on silica gel (Kieselgel 60 Merck, Germany, 0.063–0.200 mm). TLC was performed on an Alugram SIL G/UV254 (Macherey-Nagel, Düren, Germany) with UV visualization. The melting points were determined with Electrothermal Melting Point Apparatus IA6301 and are uncorrected. The ^1^H and ^13^C (with complete proton decoupling) NMR spectra were recorded on a Bruker (Karlsruhe, Germany) AMX 400 NMR instrument at 303 K. The ^1^H-NMR-spectra were recorded at 400 MHz and the ^13^C-NMR-spectra at 100 MHz. The chemical shifts in ppm were measured relative to the residual solvent signals as internal standards (CDCl_3_, ^1^H: 7.26 ppm, ^13^C: 77.1 ppm; DMSO-*d*_6_, ^1^H: 2.50 ppm, ^13^C: 39.5 ppm). Spin-spin coupling constants (*J*) are given in Hz. The high resolution mass spectra (HRMS) were registered on a Bruker Daltonics (Manning Park, Billerica, MA, USA) micrOTOF-Q II instrument using electrospray ionization (ESI). The measurements were done in positive ion mode. Interface capillary voltage: 4500 V; mass range from *m*/*z* 50 to 3000; external calibration (Electrospray Calibrant Solution, Fluka); nebulizer pressure: 0.4 Bar; flow rate: 3 µL/min; dry gas: nitrogen (4 L/min); interface temperature: 200 °C. Samples were injected into the mass spectrometer chamber from the Agilent 1260 HPLC system equipped with an Agilent (Santa Clara, CA, USA) Poroshell 120 EC-C18 (3.0 × 50 mm; 2.7 µm) column: the flow rate was 200 µL/min; and the samples were injected from the acetonitrile–water (1:1) solution and eluted in a linear gradient of acetonitrile concentrations (50→100%).

### 3.2. 9-(2,3,5-Tri-O-isobutyroyl-β-d-ribofuranosyl)-6-chloropurine *(**4**)*

Isobutyric anhydride (5.6 mL, 33.8 mmol) was added in portions to a suspension of 3 g (11.2 mmol) of inosine in 18 mL of dry pyridine. The reaction mixture was stirred for 24 h at room temperature, and then evaporated in vacuum. The residue was diluted with the mixture ethanol:water (50 mL), and the suspension was filtered. The resulting powder was washed with the mixture ethanol:water (50 mL) and dried in a vacuum dessicator over phosphorous pentoxide for 2 days. The yield was 5 g (93%) of 2′,3′,5′-tri-*O*-isobutyroylinosine as a white powder. *R*_f_ 0.39 (CH_2_Cl_2_:EtOH—98:2). ^1^H-NMR (400 MHz, CDCl_3_): δ = 1.23–1.12 (m, 18H, Me-*i*-Bu), 2.66–2.50 (m, 3H, CH-*i*-Bu), 4.40 (d, 2H, *J*_5′4′_ = 3.7 Hz, H5′), 4.45 (dt, 1H, *J*_4′5′_ = 3.7 Hz, *J*_4′3′_ = 4.6 Hz, H4′), 5.60 (dd, 1H, *J*_3′4′_ = 4.6 Hz, *J*_3′2′_ = 5.4 Hz, H3′), 5.84 (dd, 1H, *J*_2′3′_ = 5.4 Hz, *J*_2′1′_ = 5.3 Hz, H2′), 6.18 (d, 1H, *J*_1′2′_ = 5.3 Hz, H1′), 8.21 (br s, 2H, H2, H8 Hyp), 12.99 (br s, 1H, NH Hyp).

The compound 2′,3′,5′-tri-*O*-isobutyroylinosine (2.9 g, 6.05 mmol) was then dissolved in 30 mL of a DMF:dichloroethane (1:15) mixture, and thionyl chloride (1.14 mL, 15.7 mmol) was added dropwise to the mixture under intensive stirring. After stirring at 65 °C for 15 min, the reaction mixture was diluted with dichloromethane (60 mL) and washed successively with 10% sodium bicarbonate (4 × 50 mL) and water (2 × 50 mL). The organic layer was separated, dried over anhydrous Na_2_SO_4_, filtered, and evaporated in a vacuum. The residue was purified by column chromatography on silica gel (200 mL). The column was washed with dichloromethane (200 mL). The product was eluted with the system CH_2_Cl_2_:EtOH—96:4. The yield was 2.8 g (93%) as a slightly yellow syrup. *R*_f_ 0.52 (CH_2_Cl_2_:EtOH—98:2). ^1^H-NMR (400 MHz, CDCl_3_): δ = 1.09–1.23 (m, 18H, Me-*i*-Bu), 2.49–2.69 (m, 3H, CH-*i-*Bu), 4.40 (d, 2H, *J*_5′,4′_ = 3.7 Hz, H5′), 4.27 (dd, 1H, *J*_4′,5′_ = 3.7 Hz, *J*_4′,3′_ = 4.5 Hz, H4′), 5.63 (dd, 1H, *J*_3′,2′_ = 5.3 Hz, *J*_4′,3′_ = 4.5 Hz, H3′), 5.89 (t, 1H, *J*_2′,3′_ = 5.3 Hz, *J*_2′,1′_ = 5.3 Hz, H2′), 6.22 (d, 1H, *J*_1′,2′_ = 5.3 Hz, H1′), 8.30 (s, 1H, H2), 8.76 (s, 1H, H8). ^13^C-NMR (100 MHz, CDCl_3_): δ = 18.77, 18.85, 18.93, 18.99, 19.01, 19.11 (*C*H_3_-*i*-Bu), 33.80, 33.92, 34.05 (*C*H-*i*-Bu), 63.05 (C5′), 70.55 (C3′), 73.46 (C2′), 81.12 (C4′), 87.10 (C1′), 132.40 (C6), 143.57 (C5), 151.42 (C8), 151.73 (C4), 152.44 (C2), 175.51, 175.71, 176.57 (C=O). HRMS: *m*/*z* [M + H]^+^ calculated C_22_H_30_ClN_4_O_7_^+^ 497.1798, found 497.1798.

### 3.3. N*^6^*-(2-Fluorobenzyl)adenosine *(**5**)*

A mixture of **4** (200 mg, 0.4 mmol) and 2-fluorobenzylamine (0.091 mL, 0.8 mmol) was dissolved in MeCN (3 mL), and then DIPEA (0.14 mL, 0.8 mmol) was added in one portion. The solution was stirred at 70 °C. The reaction was monitored by TLC (CH_2_Cl_2_:EtOH—99.5:0.5). After 22 h, the reaction mixture was evaporated in a vacuum and the residue was diluted with methylene chloride (30 mL) and washed with water (2 × 15 mL). The organic layer was separated, dried over anhydrous Na_2_SO_4_, and evaporated in a vacuum. The residue was purified by column chromatography on silica gel. The product was eluted with CH_2_Cl_2_:EtOH—99:1. The yield was 208 mg (89%) of *N*^6^-(2-fluorobenzyl)-2′,3′,5′-tri-*O*-isobutyroyladenosine as a syrup. *R*_f_ 0.6 (CH_2_Cl_2_:EtOH—99.5:0.5). ^1^H-NMR (400 MHz, DMSO-*d*_6_): δ = 1.0–1.2 (m, 18H, Me-*i*-Bu), 2.51–2.66 (m, 3H, CH-*i*-Bu), 4.35–4.41 (m, 2H, H5′), 4.27 (ddd, 1H, *J*_4′,5′_ = 6.1 Hz, H4′), 4.76 (br s, 2H, CH_2_), 5.75 (dd, 1H, *J*_3′,2′_ = 5.4 Hz, H3′), 6.03 (dd, 1H, *J*_2′,3′_ = 5.4 Hz, *J*_2′,1′_ = 4.9 Hz, H2′), 6.21 (d, 1H, *J*_1′,2′_ = 4.9 Hz, H1′), 7.11 (td, 1H, ^3^*J*_5-6_ = 7.5 Hz, ^3^*J*_5-4_ = 7.5 Hz, ^4^*J*_5-3_ = 1.0 Hz, 5H-2-F-Ph), 7.16 (ddd, 1H, ^3^*J*_H*-*F_ = 8.2 Hz, ^3^*J*_3-4_ = 8.8 Hz, ^4^*J*_3-5_ = 1.0 Hz, 3H-2-F-Ph), 7.28 (dddd, 1H, ^4^*J*_H-F_ = 5.5 Hz, ^3^*J*_4-3_ = 8.8 Hz, ^3^*J*_4-5_= 7.5 Hz, ^4^*J*_4-6_ = 1.6 Hz, 4H-2-F-Ph), 7.32 (ddd, 1H, ^4^*J*_H-F_ = 7.5 Hz, ^3^*J*_6-5_ = 7.5 Hz, ^4^*J*_6-4_ = 1.6 Hz, 6H-2-F-Ph), 7.23 (s, 1H, H2 Ade), 8.36 (s, 1H, H8 Ade), 8.42 (1H, NH).

The resulting *N*^6^-(2-fluorobenzyl)-2′,3′,5′-tri-*O-*isobutyroyladenosine (206 mg, 0.352 mmol) was treated with 8 M MeNH_2_ in EtOH solution (4.5 mL). After 2 days, the mixture was evaporated in a vacuum and the residue was purified by column chromatography on silica gel. The column was washed with CH_2_Cl_2_:EtOH—95:5, and then eluted with CH_2_Cl_2_:EtOH—90:10 to give **5** as a white powder. The yield was 118 mg (79% for two steps). *R*_f_ 0.15 (CH_2_Cl_2_:EtOH—95:5). m.p. 194–195 °C. ^1^H-NMR (400 MHz, DMSO-*d*_6_): δ = 3.55 (ddd, 1H, *J*_5′b,5′a_ = −12.0 Hz, *J*_5′b,4′_ = 3.4 Hz, *J*_5′b,OH_ = 6.9 Hz, H5′b), 3.68 (ddd, 1H, *J*_5′a,5′b_ = −12.0 Hz, *J*_5′a,4′_ = 3.4 Hz, *J*_5′a,OH_ = 4.7 Hz, H5′a), 3.96 (q, 1H, *J*_4′,5′b_ = 3.4, *J*_4′,5′a_ = 3.4 Hz, *J*_4′,3′_ = 3.4 Hz, H4′), 4.15 (ddd, 1H, *J*_3′,4′_ = 3.4 Hz, *J*_3′,2′_ = 4.9 Hz, *J*_3′,OH_ = 4.7 Hz, H3′), 4.61 (ddd, 1H, *J*_2′,3′_ = 4.9 Hz, *J*_2′,1′_ = 6.1 Hz, *J*_2′,OH_ = 6.2 Hz, H2′), 4.77 (br s, 2H, CH_2_), 5.15 (d, 1H, *J*_OH-3′_ = 4.7 Hz, 3′OH), 5.31 (dd, 1H, *J*_OH-5′b_ = 6.9 Hz, *J*_OH-5′a_ = 4.7 Hz, 5′OH), 5.41 (d, 1H, *J*_OH-2′_ = 6.2 Hz, 2′OH), 5.90 (d, 1H, *J*_1′,2′_ = 6.1 Hz, H1′), 7.11 (td, 1H, ^3^*J*_5-6_ = 7.5 Hz, ^3^*J*_5-4_ = 7.5 Hz, ^4^*J*_5-3_ = 1.0 Hz, ^5^*J*_H-F_ < 1.0 Hz, 5H-2-F-Ph), 7.16 (ddd, 1H, ^3^*J*_H-F_ = 9.4 Hz, ^3^*J*_3-4_ = 8.2 Hz, ^4^*J*_3-5_ = 1.0 Hz, 3H-2-F-Ph), 7.26 (dddd, 1H, ^4^*J*_H-F_ = 5.5 Hz, ^3^*J*_4-3_ = 8.2 Hz, ^3^*J*_4-5_ = 7.5 Hz, ^4^*J*_4-6_ = 1.7 Hz, 4H-2-F-Ph), 7.32 (ddd, 1H, ^4^*J*_H-F_ = 6.7 Hz, ^3^*J*_6-5_ = 7.5 Hz, ^4^*J*_6-4_ = 1.7 Hz, 6H-2-F-Ph), 8.21 (s, 2H, H2, NH), 8.39 (s, 1H, H8). ^13^C-NMR (100 MHz, DMSO-*d*_6_): δ = 36.78 (CH_2_), 61.62 (C5′), 70.60 (C3′), 73.49 (C2′), 85.86 (C4′), 87.92 (C1′), 114.95 (d, ^2^*J*_C-F_ = 21.1 Hz, C3-Ph), 119.77 (C5), 124.18 (C5-Ph), 126.52 (d, ^2^*J*_C-F_ = 12.6 Hz, C1-Ph), 128.52 (d, ^3^*J*_C-F_ = 7.4 Hz, C4-Ph), 128.85 (br s, C6-Ph), 140.00 (C8), 148.59 (C4), 152.29 (C2), 154.49 (C6), 160.00 (d, ^1^*J*_C-F_ = 244.0 Hz, C2-Ph). HRMS: *m*/*z* [M + H]^+^ calculated C_17_H_19_FN_5_O_4_^+^ 376.1416, found 376.1417; *m*/*z* [M + Na]^+^ calculated C_17_H_18_FN_5_O_4_Na^+^ 398.1235, found 398.1238.

### 3.4. N*^6^*-(3-Fluorobenzyl)adenosine *(**6**)*

Following the procedure for the preparation of **5**, the condensation of 4 (200 mg, 0.4 mmol) with 3-fluorobenzylamine (0.091 mL, 0.8 mmol) in the presence of DIPEA (0.14 mL, 0.8 mmol) in MeCN (3 mL) for 22 h at 70 °C with a subsequent deblocking in 8 M MeNH_2_ in EtOH solution (4.5 mL) at room temperature gave **6** as a white powder. The overall yield was 111 mg (74%). *R*_f_ 0.15 (CH_2_Cl_2_:EtOH—95:5). m.p. 159–160 °C. ^1^H-NMR (400 MHz, DMSO-*d*_6_): δ = 3.56 (ddd, 1H, *J*_5′b,5′a_ = −12.0 Hz, *J*_5′b,4′_ = 3.4 Hz, *J*_5′b,OH_ = 6.9 Hz, H5′b), 3.67 (ddd, 1H, *J*_5′a,5′b_ = −12.0 Hz, *J*_5′a,4′_ = 3.4 Hz, *J*_5′a,OH_ = 4.7 Hz, H5′a), 3.96 (q, 1H, *J*_4′,5′b_ = 3.4 Hz, *J*_4′,5′a_ = 3.4 Hz, *J*_4′,3′_ = 3.4 Hz, H4′), 4.15 (td, 1H, *J*_3′,4′_ = 3.4 Hz, *J*_3′,2′_ = 4.7 Hz, *J*_3′,OH_ = 4.7 Hz, H3′), 4.61 (ddd, 1H, *J*_2′,3′_ = 4.7 Hz, *J*_2′,1′_ = 6.1 Hz, *J*_2′,OH_ = 6.2 Hz, H2′), 4.77 (br s, 2H, CH_2_), 5.15 (d, 1H, *J*_OH-3′_ = 4.7 Hz, 3′OH), 5.32 (dd, 1H, *J*_OH-5′b_ = 6.9 Hz, *J*_OH-5′a_ = 4.7 Hz, 5′OH), 5.41 (d, 1H, *J*_OH-2′_ = 6.2 Hz, 2′OH), 5.91 (d, 1H, *J*_1′,2′_ = 6.1 Hz, H1′), 7.03 (dd, 1H, ^3^*J*_H-F_ = 8.9 Hz, ^3^*J*_4-5_ = 8.2 Hz, 4H-3-F-Ph), 7.13 (d, 1H, ^3^*J*_H-F_ = 8.9 Hz, 2H*-*3-F-Ph), 7.18 (dd, 1H, ^3^*J*_6*-*5_ = 8.2 Hz, ^5^*J*_H*-*F_ = 2.2 Hz, 6H*-*3-F-Ph), 7.33 (td, 1H, ^4^*J*_H-F_ = 6.3 Hz, ^3^*J*_5-4_ = 8.2 Hz, ^3^*J*_5-6_ = 8.2 Hz, 5H-3-F-Ph), 8.21 (s, 1H, H2 Ade), 8.39 (s, 1H, H8 Ade), 8.46 (br s, 1H, NH). ^13^C-NMR (100 MHz, DMSO-*d*_6_): δ = 42.51 (CH_2_), 61.61 (C5′), 70.60 (C3′), 73.49 (C2′), 85.86 (C4′), 87.93 (C1′), 113.29 (d, ^2^*J*_C-F_ = 21.0 Hz, C2-Ph), 113.71 (d, ^2^*J*_C-F_ = 21.5 Hz, C4-Ph), 119.76 (C5), 123.06 (C6-Ph), 130.10 (d, ^3^*J*_C-F_ = 7.5 Hz, C5-Ph), 139.99 (C8), 143.09 (br s, C1-Ph), 148.54 (C4), 152.30 (C2), 154.44 (C6), 162.16 (d, ^1^*J*_C-F_ = 243.1 Hz, C3-Ph). HRMS: *m*/*z* [M + H]^+^ calculated C_17_H_19_FN_5_O_4_^+^ 376.1416, found 376.1418; *m/z* [M + Na]^+^ calculated C_17_H_18_FN_5_O_4_Na^+^ 398.1235, found 398.1239.

### 3.5. N*^6^*-(4-Fluorobenzyl)adenosine *(**7**)*

Following the procedure for the preparation of **5**, the condensation of **4** (200 mg, 0.4 mmol) with 4-fluorobenzylamine (0.091 mL, 0.8 mmol) in the presence of DIPEA (0.14 mL, 0.8 mmol) in MeCN (3 mL) for 22 h at 70 °C with a subsequent deblocking in 8 M MeNH_2_ in EtOH solution (4.5 mL) at room temperature gave 7 as a white powder. The overall yield was 81 mg (54%). *R*_f_ 0.15 (CH_2_Cl_2_:EtOH—95:5). mp 181–182 °C. ^1^H-NMR (400 MHz, DMSO-*d*_6_): δ = 3.55 (ddd, 1H, *J*_5′b,5′a_ = −12.0 Hz, *J*_5′b,4′_ = 3.4 Hz, *J*_5′b,OH_ = 6.9 Hz, H5′b), 3.68 (ddd, 1H, *J*_5′a,5′b_ = −12.0 Hz, *J*_5′a,4′_ = 3.4 Hz, *J*_5′a,OH_ = 4.6 Hz, H5′a), 3.96 (q, 1H, *J*_4′,5′b_ = 3.4 Hz, *J*_4′,5′a_ = 3.4 Hz, *J*_4′,3′_ = 3.4 Hz, H4′), 4.15 (ddd, 1H, *J*_3′,4′_ = 3.4 Hz, *J*_3′,2′_ = 4.7 Hz, *J*_3′,OH_ = 4.7 Hz, H3′), 4.62 (ddd, 1H, *J*_2′,3′_ = 4.7 Hz, *J*_2′,1′_ = 6.1 Hz, *J*_2′,OH_ = 6.2 Hz, H2′), 4.69 (br s, 2H, CH_2_), 5.15 (d, 1H, *J*_OH-3′_ = 4.7 Hz, 3′OH), 5.32 (dd, 1H, *J*_OH-5′b_ = 6.9 Hz, *J*_OH-5′a_ = 4.6 Hz, 5′OH), 5.41 (d, 1H, *J*_OH-2′_ = 6.2 Hz, 2′OH), 5.89 (d, 1H, *J*_1′,2′_ = 6.1 Hz, H1′), 7.11 (t, 2H, ^3^*J*_H-F_ = 8.9 Hz, ^3^*J*_3-2_ = 8.9 Hz, 3H-4-F-Ph, 5H*-*4-F-Ph), 7.37 (dd, 2H, ^3^*J*_2-3_ = 8.9, ^4^*J*_H-F_ = 6.0 Hz, 2H-4-F-Ph, 6H*-*4-F-Ph), 8.21 (s, 1H, H2 Ade), 8.37 (s, 1H, H8 Ade), 8.43 (br s, 1H, NH). ^13^C-NMR (100 MHz, DMSO-*d*_6_): δ = 42.21 (CH_2_), 61.62 (C5′), 70.61 (C3′), 73.48 (C2′), 85.87 (C4′), 87.93 (C1′), 114.87 (d, ^2^*J*_C-F_ = 21.2 Hz, C3-Ph, C5-Ph), 119.74 (C5), 129.05 (d, ^3^*J*_C-F_ = 7.5 Hz, C2-Ph, C6-Ph), 136.15 (C1-Ph), 139.91 (C8), 148.52 (C4), 152.30 (C2), 154.43 (C6), 161.07 (d, ^1^*J*_C-F_ = 241.9 Hz, C4-Ph). HRMS: *m/z* [M + H]^+^ calculated C_17_H_19_FN_5_O_4_^+^ 376.1416, found 376.1407; *m*/*z* [M + Na]^+^ calculated C_17_H_18_FN_5_O_4_Na^+^ 398.1235, found 398.1227.

### 3.6. N*^6^*-(2,6-Difluorobenzyl)adenosine *(**8**)*

Following the procedure for the preparation of **5**, the condensation of **4** (191 mg, 0.384 mmol) with 2,6-difluorobenzylamine (0.092 mL, 0.77 mmol) in the presence of DIPEA (0.134 mL, 0.77 mmol) in MeCN (2.5 mL) for 11 h at 70 °C with a subsequent deblocking in 8 M MeNH_2_ in EtOH solution (2 mL) at room temperature gave **8** as a white powder. The overall yield was 129 mg (85%). *R*_f_ 0.15 (CH_2_Cl_2_:EtOH—95:5). m.p. 192–194 °C. ^1^H-NMR (400 MHz, DMSO-*d*_6_): δ = 3.56 (ddd, 1H, *J*_5′b,5′a_ = −12.0 Hz, *J*_5′b,4′_ = 3.5 Hz, *J*_5′b,OH_ = 6.8 Hz, H5′b), 3.68 (ddd, 1H, *J*_5′a,5′b_ = −12.0 Hz, *J*_5′a,4′_ = 3.5 Hz, *J*_5′a,OH_ = 4.7 Hz, H5′a), 3.97 (td, 1H, *J*_4′,5′b_ = 3.5 Hz, *J*_4′,5′a_ = 3.5 Hz, *J*_4′,3′_ = 3.3 Hz, H4′), 4.15 (ddd, 1H, *J*_3′,4′_ = 3.3 Hz, *J*_3′,2′_ = 5.2 Hz, *J*_3′,OH_ = 4.6 Hz, H3′), 4.61 (td, 1H, *J*_2′,3′_ = 5.2 Hz, *J*_2′,1′_ = 6.1 Hz, *J*_2′,OH_ = 6.1 Hz, H2′), 4.80 (br s, 2H, CH_2_), 5.15 (d, 1H, *J*_OH-3′_ = 4.6 Hz, 3′OH), 5.31 (dd, 1H, *J*_OH-5′b_ = 7.0 Hz, *J*_OH-5′a_ = 4.7 Hz, 5′OH), 5.41 (d, 1H, *J*_OH-2′_ = 6.1 Hz, 2′OH), 5.89 (d, 1H, *J*_1′,2′_ = 6.1 Hz, H1′), 7.05 (dd, 2H, ^3^*J*_H-F_ =7.6 Hz, *J*_m-H-p-H_ = 8.9 Hz, *m*-H-2,6-di-F-Ph), 7.37 (tt, 1H, *J*_p-H-m-H_ =8.9 Hz, ^4^*J*_H-F_ = 6.0 Hz, *p-*H-2,6-di-F-Ph), 8.19 (br s, 1H, NH), 8.24 (s, 1H, H8 Ade), 8.36 (s, 1H, H2 Ade). ^13^C-NMR (100 MHz, DMSO-*d*_6_): δ = 32.17 (CH_2_), 61.61 (C5′), 70.59 (C3′), 73.50 (C2′), 85.83 (C4′), 87.93 (C1′), 111.38 (d, ^2^*J*_C-F_ = 23.6 Hz, C3-Ph, C5-Ph), 114.44 (t, ^2^*J*_C-F_ = 17.6 Hz, C1-Ph), 119.70 (C5), 129.60 (C4-Ph), 139.85 (C8), 148.64 (C4), 152.17 (C2), 154.16 (C6), 161.23 (d, ^1^*J*_C-F_ = 248.0 Hz, C2-Ph, C6-Ph). HRMS: *m/z* [M + H]^+^ calculated C_17_H_18_F_2_N_5_O_4_^+^ 394.1321, found 394.1325; *m*/*z* [M + Na]^+^ calculated C_17_H_17_F_2_N_5_O_4_Na^+^ 416.1141, found 416.1143.

### 3.7. N*^6^*-(2-Trifluoromethylbenzyl)adenosine *(**9**)*

Following the procedure for the preparation of **5**, the condensation of **4** (213 mg, 0.43 mmol) with 2-trifluoromethylbenzylamine (0.12 mL, 0.86 mmol) in the presence of DIPEA (0.15 mL, 0.86 mmol) in MeCN (3 mL) for 10 h at 70 °C with a subsequent deblocking in 8 M MeNH_2_ in EtOH solution (3 mL) at room temperature gave **9** as a white powder. The overall yield was 168 mg (92%). *R*_f_ 0.13 (CH_2_Cl_2_:EtOH—95:5). m.p. 204–205 °C. ^1^H-NMR (400 MHz, DMSO-*d*_6_): δ = 3.56 (ddd, 1H, *J*_5′b,5′a_ = −12.0 Hz, *J*_5′b,4′_ = 3.5 Hz, *J*_5′b,OH_ = 6.9 Hz, H5′b), 3.68 (ddd, 1H, *J*_5′a,5′b_ = −12.0 Hz, *J*_5′a,4′_ = 3.5 Hz, *J*_5′a,OH_ = 4.7 Hz, H5′a), 3.97 (q, 1H, *J*_4′,5′b_ = 3.5 Hz, *J*_4′,5′a_ = 3.5 Hz, *J*_4′,3′_ = 3.5 Hz, H4′), 4.16 (ddd, 1H, *J*_3′,4′_ = 3.5 Hz, *J*_3′,2′_ = 5.2 Hz, *J*_3′,OH_ = 4.7 Hz, H3′), 4.63 (ddd, 1H, *J*_2′,3′_ = 5.2 Hz, *J*_2′,1′_ = 6.1 Hz, *J*_2′,OH_ = 6.2 Hz, H2′), 4.93 (br s, 2H, CH_2_), 5.15 (d, 1H, *J*_OH-3′_ = 4.7 Hz, 3′OH), 5.29 (dd, 1H, *J*_OH-5′b_ = 6.9 Hz, *J*_OH-5′a_ = 4.7 Hz, 5′OH), 5.41 (d, 1H, *J*_OH-2′_ = 6.2 Hz, 2′OH), 5.91 (d, 1H, *J*_1′,2′_ = 6.1 Hz, H1′), 7.40–7.75 (m, 4H, Ph), 8.19 (s, 1H, H8), 8.42 (s, 1H, H2), 8.45 (br s, 1H, NH). ^13^C-NMR (100 MHz, DMSO-*d*_6_): δ = 39.52 (CH_2,_ overlapping with the solvent peak), 61.62 (C5′), 70.62 (C3′), 73.51 (C2′), 85.89 (C4′), 87.92 (C1′), 119.83 (C5), 123.24 (C2 Ph), 125.77 (C6 Ph), 125.95 (q, *J*_C-F_ = 30.7 Hz, CF_3_), 127.03 (C3 Ph), 127.54 (C4 Ph), 132.55 (C5 Ph), 138.10 (C1 Ph), 140.17 (C8), 146.68 (C4), 152.38 (C2), 154.52 (C6). HRMS: *m*/*z* [M + H]^+^ calculated C_18_H_19_F_3_N_5_O_4_^+^ 426.1384, found 426.1387; *m*/*z* [M + Na]^+^ calculated C_18_H_18_F_3_N_5_O_4_Na^+^ 448.1203, found 448.1210.

### 3.8. N*^6^*-(3-Trifluoromethylbenzyl)adenosine *(**10**)*

Following the procedure for the preparation of **5**, the condensation of **4** (137 mg, 0.276 mmol) with 3-trifluoromethylbenzylamine (0.08 mL, 0.55 mmol) in the presence of DIPEA (0.096 mL, 0.55 mmol) in MeCN (2 mL) for 20 h at 70 °C with a subsequent deblocking in 8 M MeNH_2_ in EtOH solution (2 mL) at room temperature gave **10** as a white powder. The overall yield was 59 mg (50%). *R*_f_ 0.11 (CH_2_Cl_2_:EtOH—95:5). m.p. 139–140 °C. ^1^H-NMR (400 MHz, DMSO-*d*_6_): δ = 3.55 (ddd, 1H, *J*_5′b,5′a_ = −12.0 Hz, *J*_5′b,4′_ = 3.6 Hz, *J*_5′b,OH_ = 7.0 Hz, H5′b), 3.68 (ddd, 1H, *J*_5′a,5′b_ = −12.0 Hz, *J*_5′a,4′_ = 3.6 Hz, *J*_5′a,OH_ = 4.7 Hz, H5′a), 3.97 (q, 1H, *J*_4′,5′b_ = 3.6 Hz, *J*_4′,5′a_ = 3.6 Hz, *J*_4′,3′_ = 3.6 Hz, H4′), 4.16 (ddd, 1H, *J*_3′,4′_ = 3.6 Hz, *J*_3′,2′_ = 5.2 Hz, *J*_3′,OH_ = 4.7 Hz, H3′), 4.62 (ddd, 1H, *J*_2′,3′_ = 5.2 Hz, *J*_2′,1′_ = 6.1 Hz, *J*_2′,OH_ = 6.2 Hz, H2′), 4.80 (br s, 2H, CH_2_), 5.14 (d, 1H, *J*_OH-3′_ = 4.7 Hz, 3′OH), 5.30 (dd, 1H, *J*_OH-5′b_ = 7.0 Hz, *J*_OH-5′a_ = 4.7 Hz, 5′OH), 5.40 (d, 1H, *J*_OH-2′_ = 6.2 Hz, 2′OH), 5.90 (d, 1H, *J*_1′,2′_ = 6.1 Hz, H1′), 7.5–7.75 (m, 4H, Ph), 8.21 (s, 1H, H8), 8.39 (s, 1H, H2), 8.53 (br s, 1H, NH). ^13^C-NMR (100 MHz, DMSO-*d*_6_): δ = 42.60 (CH_2_), 61.63 (C5′), 70.63 (C3′), 73.54 (C2′), 85.89 (C4′), 87.97 (C1′), 119.79 (C5), 123.41 (C4 Ph), 123.69 (C2 Ph), 125.64 (C6 Ph), 128.96 (q, *J*_C-F_ = 31.4 Hz, CF_3_), 129.30 (C5 Ph), 131.32 (C3 Ph), 140.08 (C8), 141.54 (C1 Ph), 148.58 (C4), 152.33 (C2), 154.43 (C6). HRMS: *m/z* [M + H]^+^ calculated C_18_H_19_F_3_N_5_O_4_^+^ 426.1384, found 426.1384; *m*/*z* [M + Na]^+^ calculated C_18_H_18_F_3_N_5_O_4_Na^+^ 448.1203, found 448.1204

### 3.9. N*^6^*-(4-Trifluoromethylbenzyl)adenosine *(**11**)*

Following the procedure for the preparation of **5**, the condensation of **4** (190 mg, 0.382 mmol) with 4-trifluoromethylbenzylamine (0.11 mL, 0.76 mmol) in the presence of DIPEA (0.132 mL, 0.76 mmol) in MeCN (3 mL) for 10 h at 70 °C with a subsequent deblocking in 8 M MeNH_2_ in EtOH solution (3 mL) at room temperature gave **11** as a white powder. The overall yield was 160 mg (98%). *R*_f_ 0.09 (CH_2_Cl_2_:EtOH—95:5). m.p. 152–153 °C. ^1^H-NMR (400 MHz, DMSO-*d*_6_): δ = 3.55 (ddd, 1H, *J*_5′b,5′a_ = −12.0 Hz, *J*_5′b,4′_ = 3.6 Hz, *J*_5′b,OH_ = 6.9 Hz, H5′b), 3.68 (ddd, 1H, *J*_5′a,5′b_ = −12.0 Hz, *J*_5′a,4′_ = 3.6 Hz, *J*_5′a,OH_ = 4.7 Hz, H5′a), 3.97 (q, 1H, *J*_4′,5′b_ = 3.6, *J*_4′,5′a_ = 3.6 Hz, *J*_4′,3′_ = 3.6 Hz, H4′), 4.16 (ddd, 1H, *J*_3′,4′_ = 3.6 Hz, *J*_3′,2′_ = 5.2 Hz, *J*_3′,OH_ = 4.7 Hz, H3′), 4.62 (ddd, 1H, *J*_2′,3′_ = 5.2 Hz, *J*_2′,1′_ = 6.1 Hz, *J*_2′,OH_ = 6.2 Hz, H2′), 4.80 (br s, 2H, CH_2_), 5.14 (d, 1H, *J*_OH-3′_ = 4.7 Hz, 3′OH), 5.30 (dd, 1H, *J*_OH-5′b_ = 6.9 Hz, *J*_OH-5′a_ = 4.7 Hz, 5′OH), 5.40 (d, 1H, *J*_OH-2′_ = 6.2 Hz, 2′OH), 5.90 (d, 1H, *J*_1′,2′_ = 6.1 Hz, H1′), 7.5–7.75 (m, 4H, Ph), 8.20 (s, 1H, H8), 8.39 (s, 1H, H2), 8.51 (br s, 1H, NH). ^13^C-NMR (100 MHz, DMSO-*d*_6_): δ = 42.65 (CH_2_), 61.60 (C5′), 70.59 (C3′), 73.49 (C2′), 85.86 (C4′), 87.91 (C1′), 119.79 (C5), 122.98 (C4 Ph), 125.07 (C3 Ph, C5 Ph), 127.48 (q, *J*_C-F_ = 30.7 Hz, CF_3_), 127.67 (C2 Ph, C6 Ph), 140.02 (C8), 144.92 (C1 Ph), 148.59 (C4), 152.29 (C2), 154.42 (C6). HRMS: *m*/*z* [M + H]^+^ calculated C_18_H_19_F_3_N_5_O_4_^+^ 426.1384, found 426.1383; *m*/*z* [M + Na]^+^ calculated C_18_H_18_F_3_N_5_O_4_Na^+^ 448.1203, found 448.1203.

### 3.10. Antiviral Assay Against EV71 in RD Cells

An EV71 BrCr laboratory-adapted strain was used at a low multiplicity of infection (MOI) in a standardized antiviral assay [31]. Briefly, freshly harvested rhadbdosarcoma (RD) cells were seeded in a 96-well plate (2 × 10^4^ cells/well) and incubated at 37 °C in 5% CO_2_. The next day, a serial dilution of the compounds was prepared in assay medium and added to the RD cells. Then, the cells were supplemented with the viral suspension. The assay plates were incubated until full virus–induced cell death was observed in the untreated, infected controls (3–4 days post-infection). Subsequently, the antiviral effect was quantified using a colorimetric readout with 3-(4,5-dimethylthiazol-2-yl)-5-(3-carboxymethoxyphenyl)-2-(4-sulfophenyl)-2*H*-tetrazolium/phenazine methosulfate (MTS/PMS method), and the concentration of compound at which a 50% inhibition of virus-induced cell death would be observed (EC_50_) was calculated from the antiviral dose-response curves. A similar assay setup was used to determine the adverse effect of the compound on uninfected, treated cells for the calculation of the CC_50_ (concentration of compound that reduces overall cell health with 50% as determined by the MTS/PMS method). The selectivity index (SI) was calculated as a ratio of EC_50_/CC_50_.

## 4. Conclusions

We reported here the antiviral profile of a class of analogues of the cytokinin nucleoside BAPR, previously described by our groups as a potent and selective inhibitor of EV71 replication. Interestingly, we showed that the replacement of hydrogen with fluorine atoms or a trifluoromethyl group in the aromatic moiety of BAPR overall increased its SI. In particular, the least successful analogue (compound **7**) with the addition of one fluorine atom at position 4 of the BAPR phenyl showed a 1.25-times increase of SI, whereas the best analogue of the class (compound **10**) with a trifluoromethyl at position 3 exhibited an SI that was 230 times higher than BAPR. Fluorinated analogues of natural substances are of interest, since often the biological activity of the parent compounds is improved. However, the tremendous gain in selectivity reported here represents one of the most spectacular examples of structure optimization of a lead natural compound by introducing fluoro- or trifluoromethyl groups into an aromatic moiety.

## Figures and Tables

**Table 1:** Stability of *O*-acyl protecting groups under different deblocking conditions.

Substrate	*O*-Deacylation Conditions, 20 °C ^a^	*t*_½_. h	Complete *O*-Deacylation, h
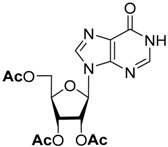	CH_3_NH_2_/C_2_H_5_OH (4M)	0.25	2
	NH_3_/MeOH (4M)	1	5
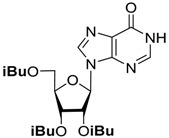	CH_3_NH_2_/C_2_H_5_OH (4M)	3	15
	NH_3_/MeOH (4M)	15	75
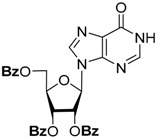	CH_3_NH_2_/C_2_H_5_OH (4M)	6	26
	NH_3_/MeOH (4M)	19	96

^a^ The reagent was used in at least 400-fold excess.

**Table 2:**
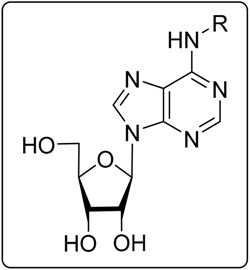
Antiviral effect of *N*^6^-substituted adenosines on the replication of the EV71 strain BrCr in RD cells.

No.	Compound Name	Substituent (R)	CC_50_ ± SD ^a,b^	EC_50_ ± SD ^a,b^	SI ^с^
**1**	*N*^6^-benzyladenosine (**BAPR**)	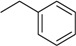	4.3 ± 1.6	0.28 ± 0.05	15
**2**	*N*^6^-isopenthenyladenosine (**iPR**)	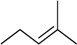	6.0 ± 0.6	1.0 ± 0.2	6.0
**3**	*N*^6^*-*furfuryladenosine (**KINR**)	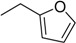	7.8 ± 3.4	1.4 ± 0.3	5.6
**5**	*N*^6^-(2-fluorobenzyl) adenosine	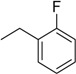	13.3 ± 3.7	0.30 ± 0.05	44
**6**	*N*^6^-(3-fluorobenzyl) adenosine	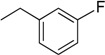	6.2 ± 1.8	0.24 ± 0.09	26
**7**	*N*^6^-(4-fluorobenzyl) adenosine	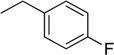	2.7 ± 0.9	0.14 ± 0.05	19
**8**	*N*^6^-(2,6-difluorobenzyl) adenosine	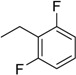	>254	0.21 ± 0.01	>1210
**9**	*N*^6^-(2-trifluoromethylbenzyl) adenosine	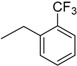	>235	1.0 ± 0.1	>235
**10**	*N*^6^-(3-trifluoromethylbenzyl) adenosine	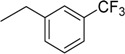	>235	0.068 ± 0.001	>3456
**11**	*N*^6^-(4-trifluoromethylbenzyl) adenosine	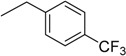	>235	1.0 ± 0.1	>235

^a^ All values are in μM and are based on at least three independent dose-response curves; ^b^ On rhabdomyosarcoma (RD) cells; ^c^ Selectivity Index (SI); SI = CC_50_/EC_50_; SD, standard deviation.

## Supplementary Materials

Supplementary Materials are available online.
