# Supplementary material for: Fluorination of Naturally Occurring N6-Benzyladenosine Remarkably Increased Its Antiviral Activity and Selectivity
Source: Molecules. 2017 Jul 20;22(7):1219. doi: 10.3390/molecules22071219 (PMC6152005; doi:10.3390/molecules22071219)
Supplement: Supplementary file 1 [file molecules-22-01219-s001.pdf]

Fluorination of naturally occurring N^6-benzyladenosine remarkably increased its antiviral activity and selectivity

(Supplementary material)

Vladimir E. Oslovsky[1], Mikhail S. Drenichev[1], Liang Sun[2], Nikolay N. Kurochkin[1], Vladislav E. Kunetsky[1], Carmen Mirabelli[2], Johan Neyts[2], Pieter Leyssen[2], Sergey N. Mikhailov[1] *

[1] *Engelhardt Institute of Molecular Biology, Russian Academy of Sciences, 119991, Vavilov Str. 32, Moscow, Russian Federation.*

[2] *KU Leuven - University of Leuven, Department of Microbiology and Immunology, Laboratory for Virology and Chemotherapy, Rega Institute for Medical Research, Minderbroedersstraat 10, 3000 Leuven, Belgium.*

* Corresponding author.

E-mail address: smikh@eimb.ru (S.N. Mikhailov).

^{1}H and ^{13}C (with complete proton decoupling) NMR spectra were recorded on Bruker AMX 400 NMR instrument at 303 K relative to the residual solvent signals as internal standards ($CDCl_3$, 1H: δ = 7.26, 13C: δ = 77.16; DMSO-d_6, 1H: δ = 2.50, 13C: δ = 39.52; CD_3OD, 1H: δ = 3.31, 13C: δ = 49.00). ^{1}H-NMR-spectra were recorded at 400 MHz and ^{13}C-NMR-spectra at 100 MHz.

High-resolution mass spectra (HRMS) were registered on a Bruker Daltonics micrOTOF-Q II instrument using electrospray ionization (ESI). The measurements were done in a positive ion mode. Interface capillary voltage: 4500 V; mass range from m/z 50 to 3000; external calibration (Electrospray Calibrant Solution, Fluka); nebulizer pressure: 0.4 Bar; flow rate: 3 μL/min; dry gas: nitrogen (4L/min); interface temperature: 200°C.

Samples were injected in to the mass spectrometer chamber from the Agilent 1260 HPLC system equipped with Agilent Poroshell 120 EC-C18 (3.0 × 50 mm; 2,7 μm) column; flow rate 200 μL/min; samples were injected from the acetonitrile-water (1:1) solution and were eluted in a linear gradient of acetonitrile concentrations (50 → 100%).

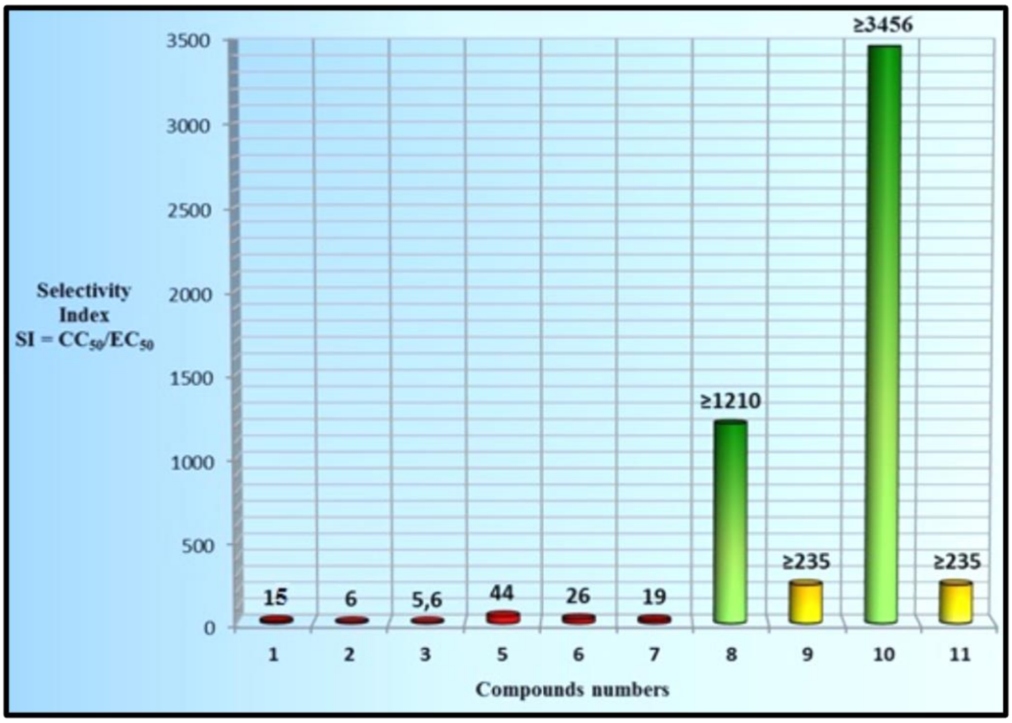

Alteration of selectivity index of *N*[6]-benzyladenosine derivatives according to the modification on the phenyl ring

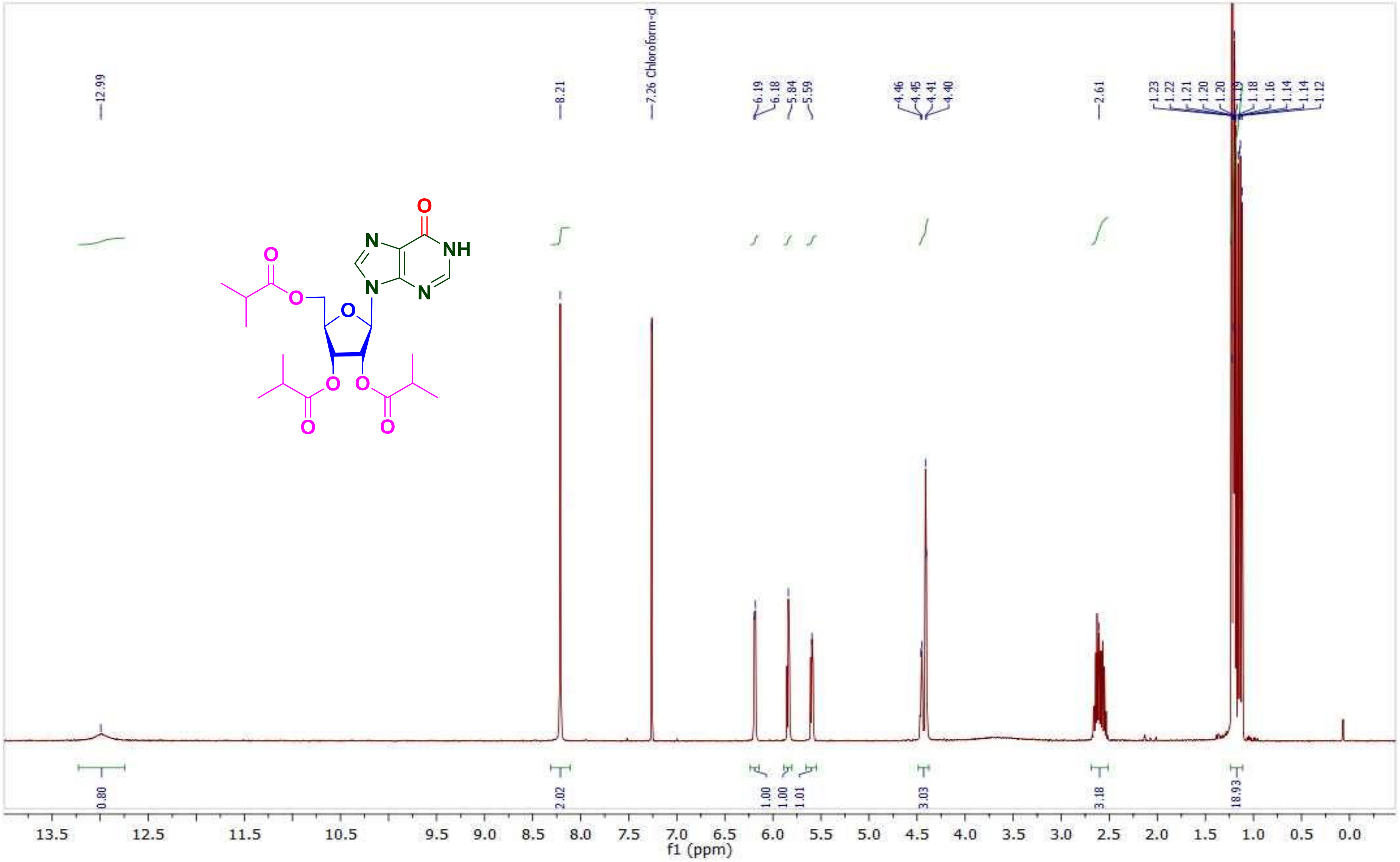

^{1}H-NMR-spectrum (400 MHz) of 2′,3′,5′-tri-O-isobutyroylinosine in $CDCl_3$ at 303 K

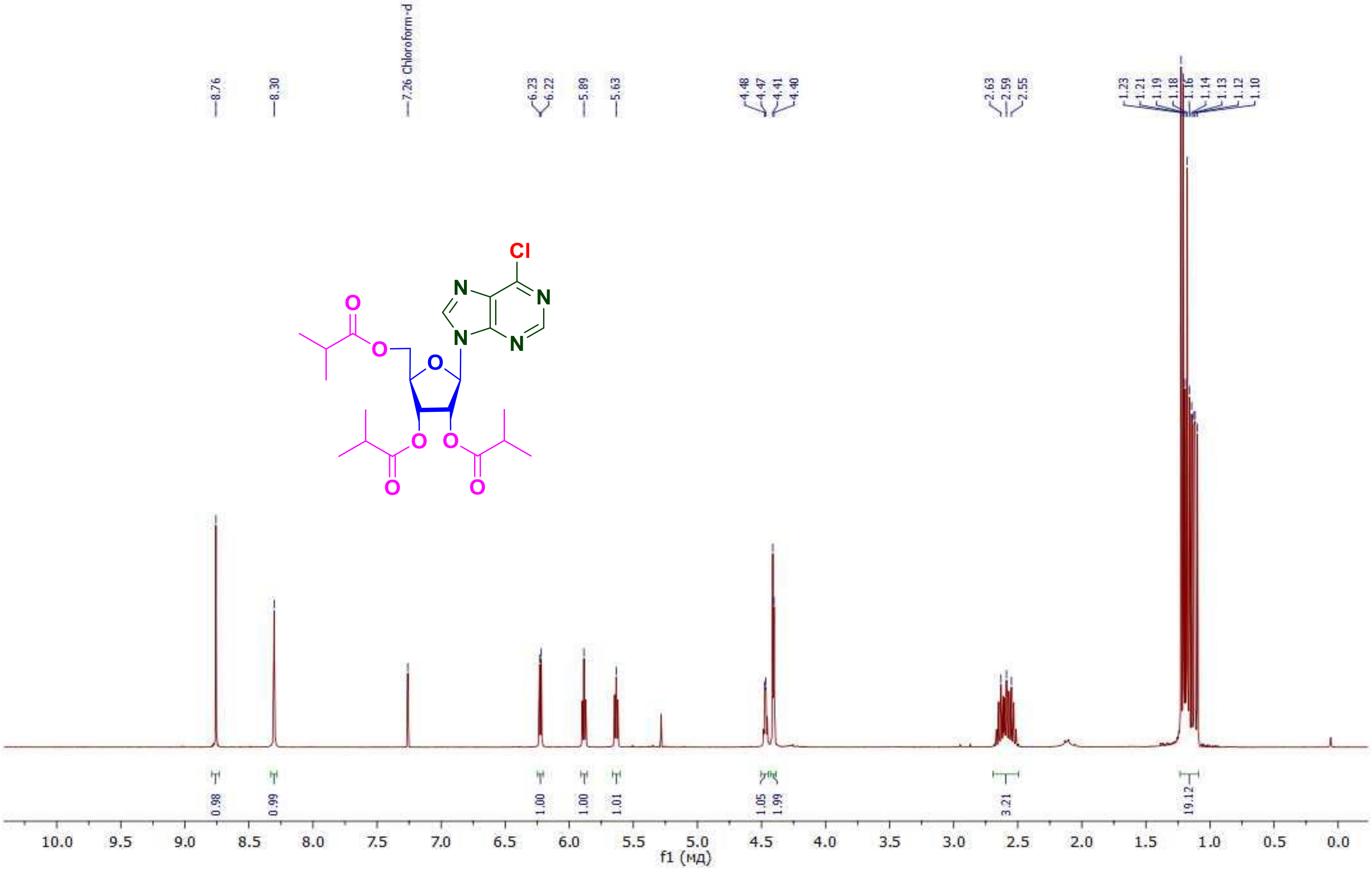

^{1}H-NMR-spectrum (400 MHz) of 6-chloro-2′,3′,5′-tri-O-isobutyroyladenosine (**4**) in $CDCl_3$ at 303 K

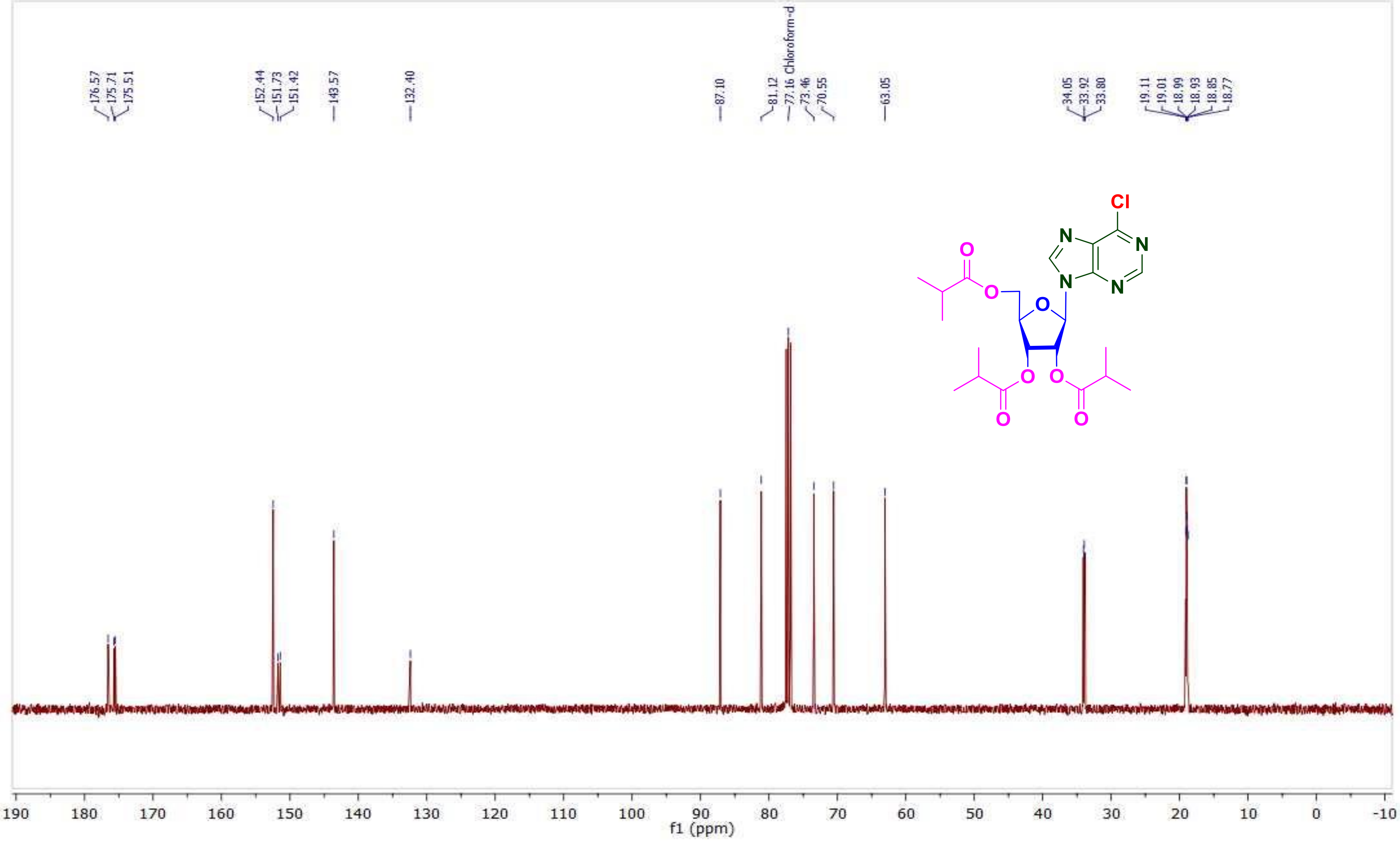

^{13}C-NMR-spectrum (100 MHz) of 6-chloro-2′,3′,5′-tri-O-isobutyroyladenosine (**4**) in $CDCl_3$ at 303 K

Acquisition Parameter

Source Type	ESI	Ion Polarity	Positive	Set Nebulizer	0.4 Bar
Focus	Active	Set Capillary	4500 V	Set Dry Heater	180 °C
Scan Begin	50 m/z	Set End Plate Offset	-500 V	Set Dry Gas	6.0 l/min
Scan End	3000 m/z	Set Charging Voltage	2000 V	Set Divert Valve	Source
		Set Corona	0 nA	Set APCI Heater	0 °C

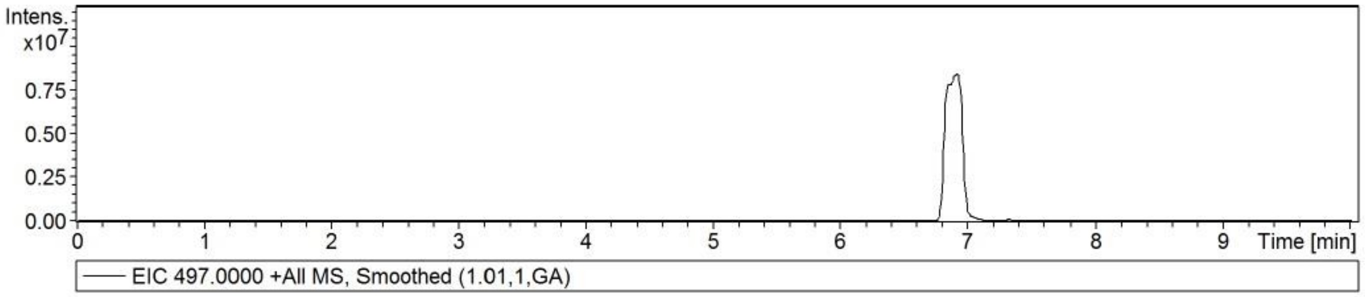

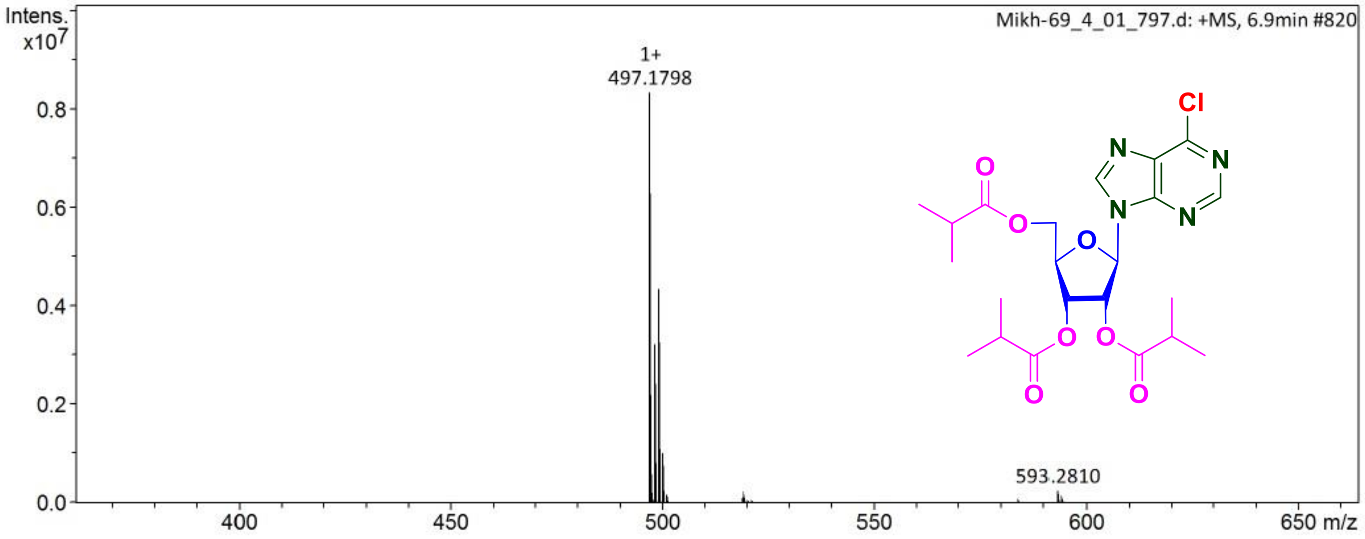

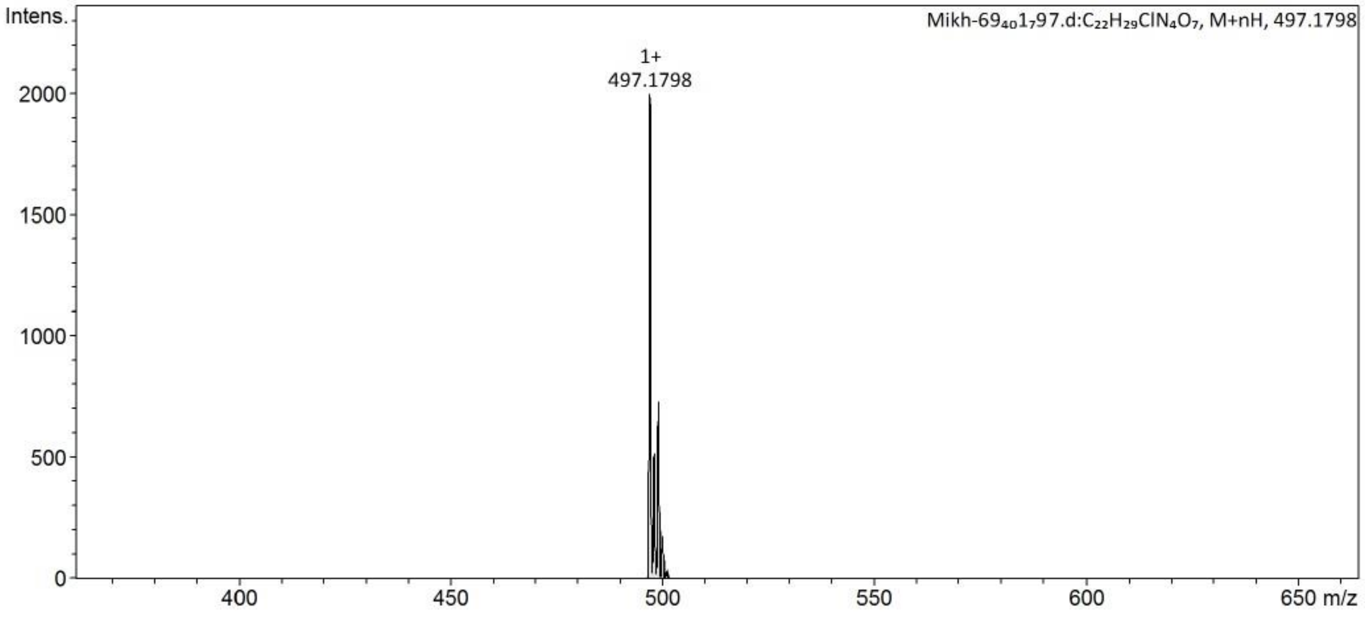

High-resolution mass spectrum (HRMS) of 6-chloro-2′,3′,5′-tri-O-isobutyroyladenosine (**4**)

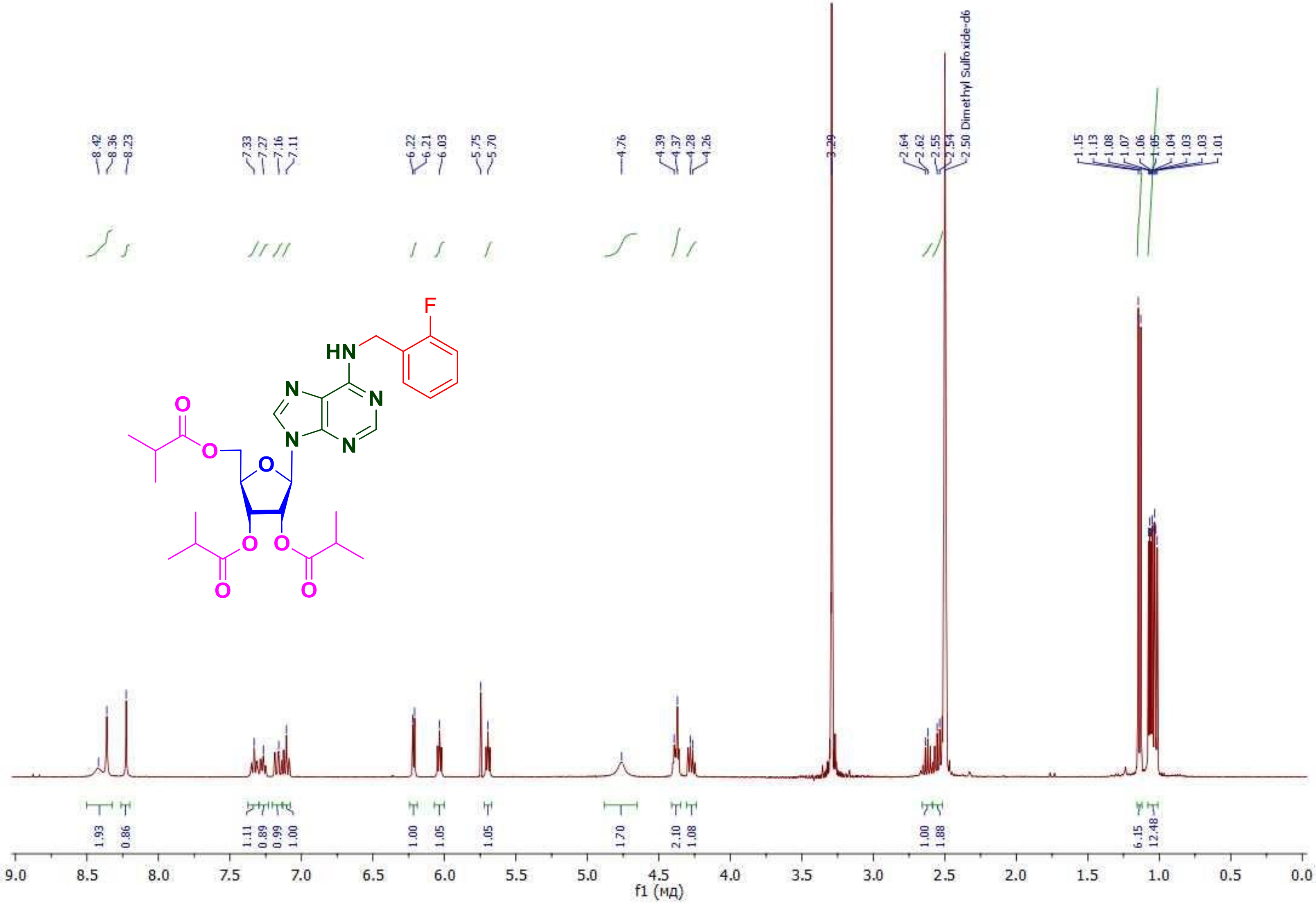

^{1}H-NMR-spectrum (400 MHz) of *N*[6]-(2-fluorobenzyl)-2′,3′,5′-tri-O-isobutyroyladenosine in DMSO-d_6 at 303 K

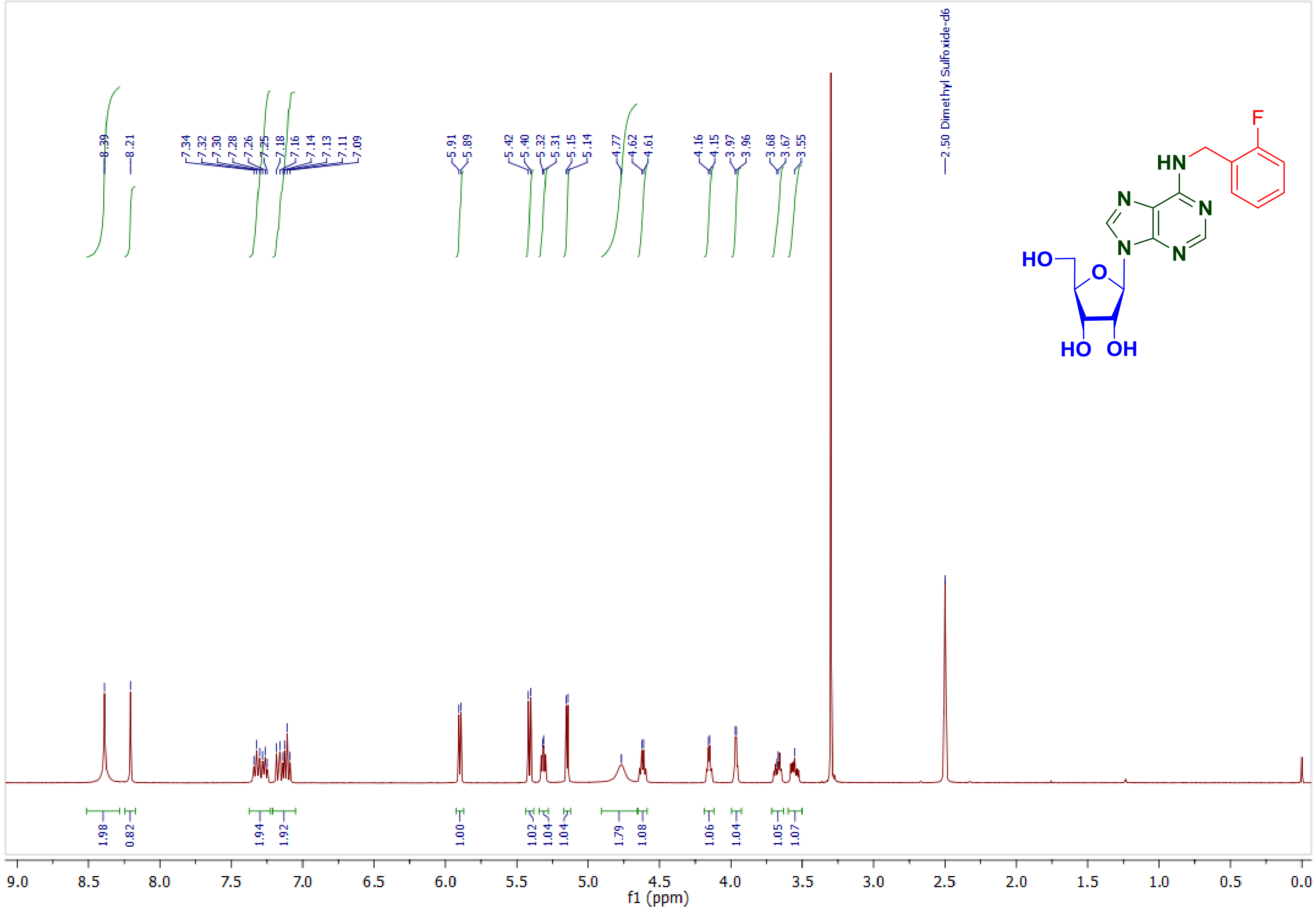

^{1}H-NMR-spectrum (400 MHz) of N^6-(2-fluorobenzyl)-adenosine (**5**) in DMSO-d_6 at 303 K

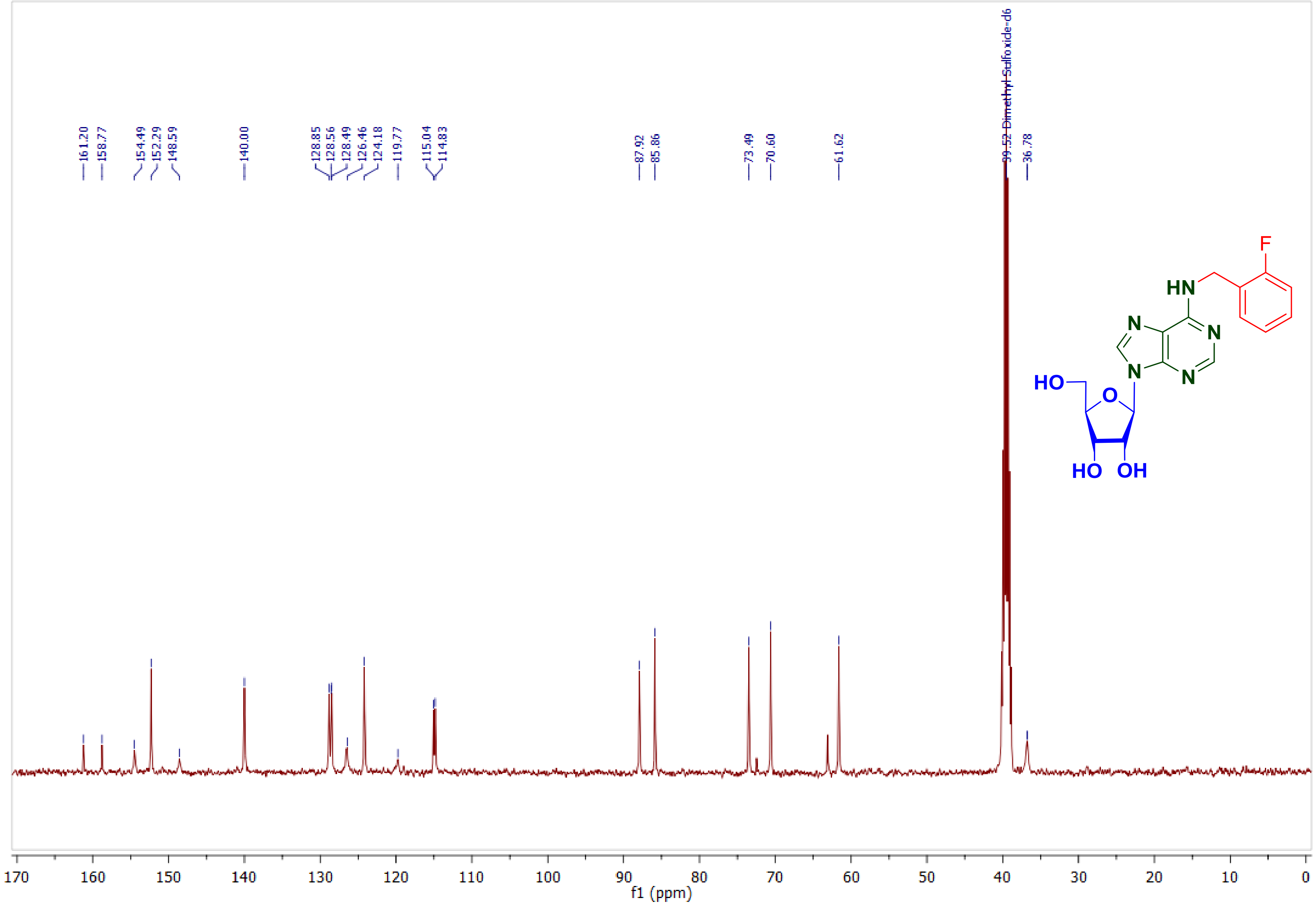

^{13}C-NMR-spectrum (100 MHz) of *N*^{6}-(2-fluorobenzyl)-adenosine (**5**) in DMSO-d_6 at 303 K

Acquisition Parameter

Source Type	ESI	Ion Polarity	Positive	Set Nebulizer	0.4 Bar
Focus	Active	Set Capillary	4500 V	Set Dry Heater	200 °C
Scan Begin	50 m/z	Set End Plate Offset	-500 V	Set Dry Gas	4.0 l/min
Scan End	3000 m/z	Set Charging Voltage	2000 V	Set Divert Valve	Source
		Set Corona	0 nA	Set APCI Heater	0 °C

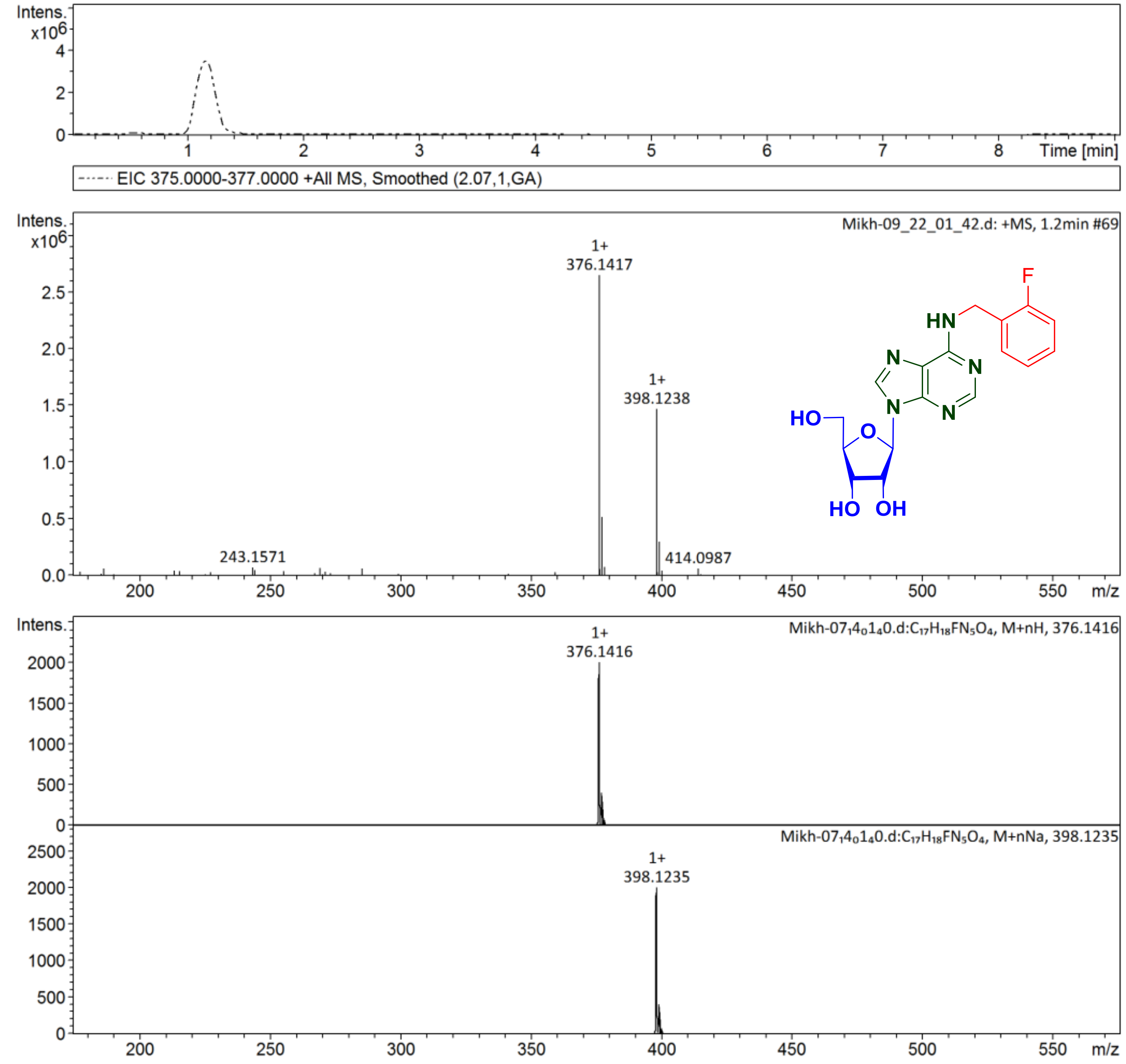

High-resolution mass spectrum (HRMS) of N^6-(2-fluorobenzyl)-adenosine (**5**)

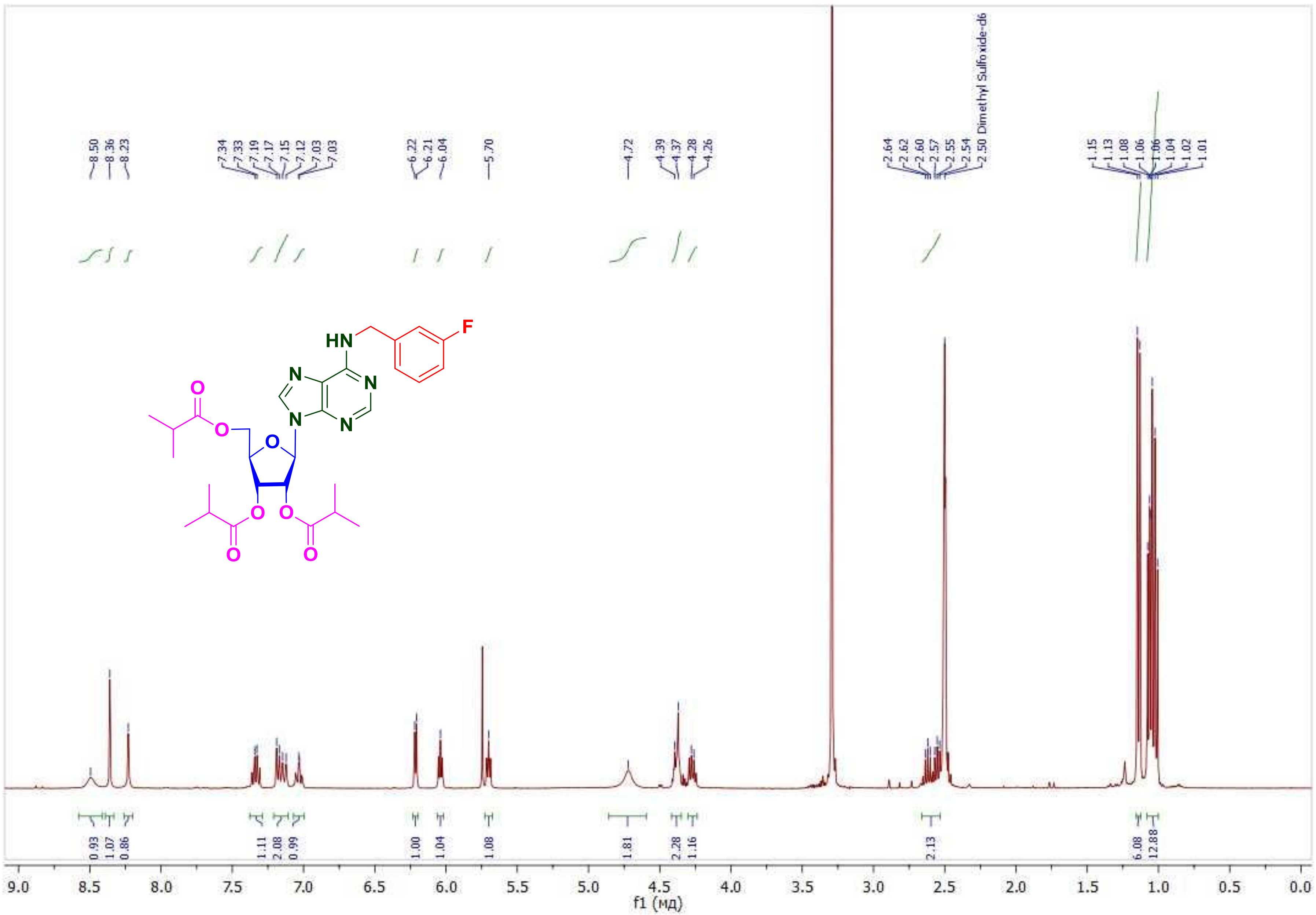

^{1}H-NMR-spectrum (400 MHz) of N^6-(3-fluorobenzyl)-2′,3′,5′-tri-O-isobutyroyladenosine in DMSO-d_6 at 303 K

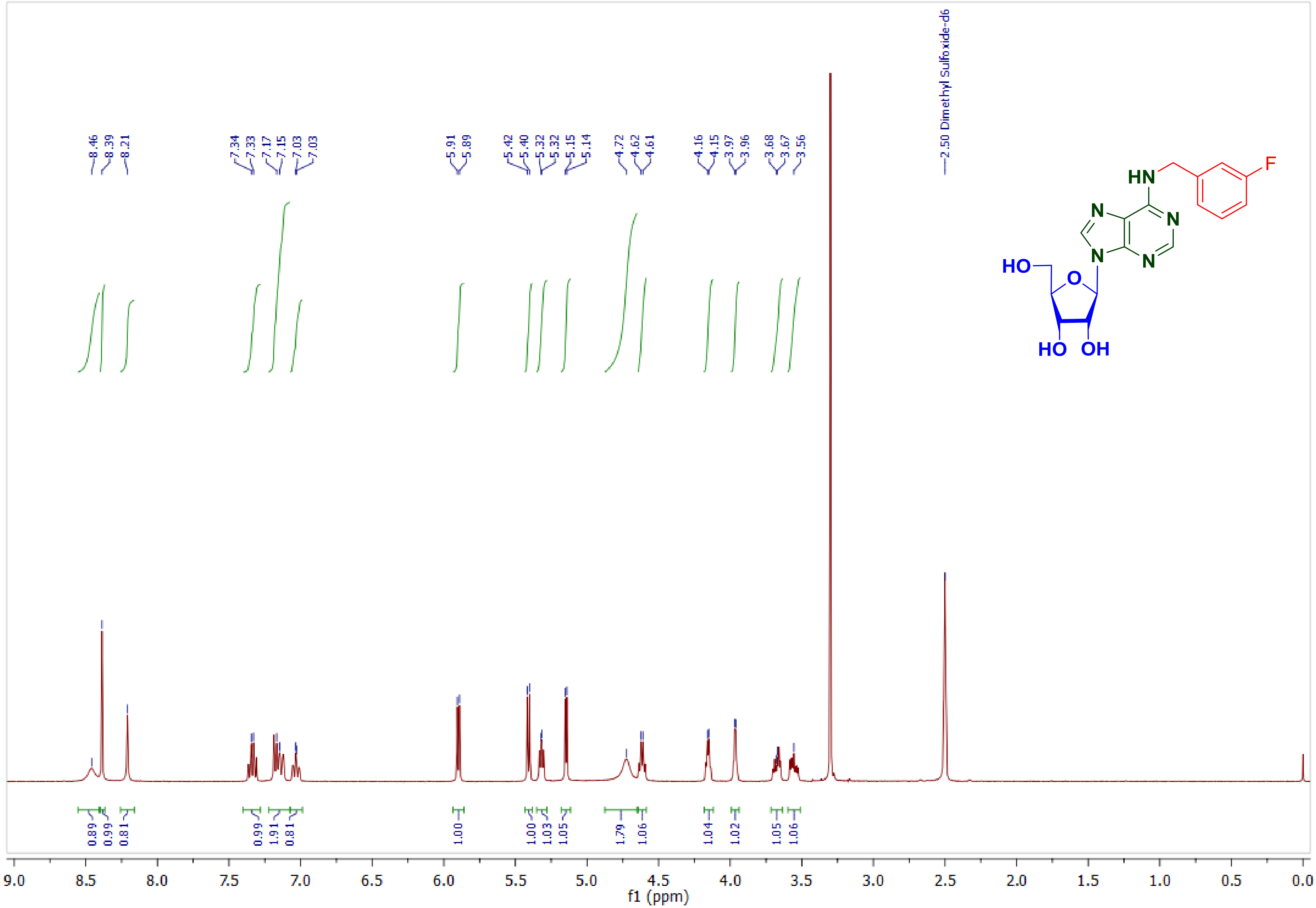

^{1}H-NMR-spectrum (400 MHz) of N^6-(3-fluorobenzyl)-adenosine (**6**) in DMSO-d_6 at 303 K

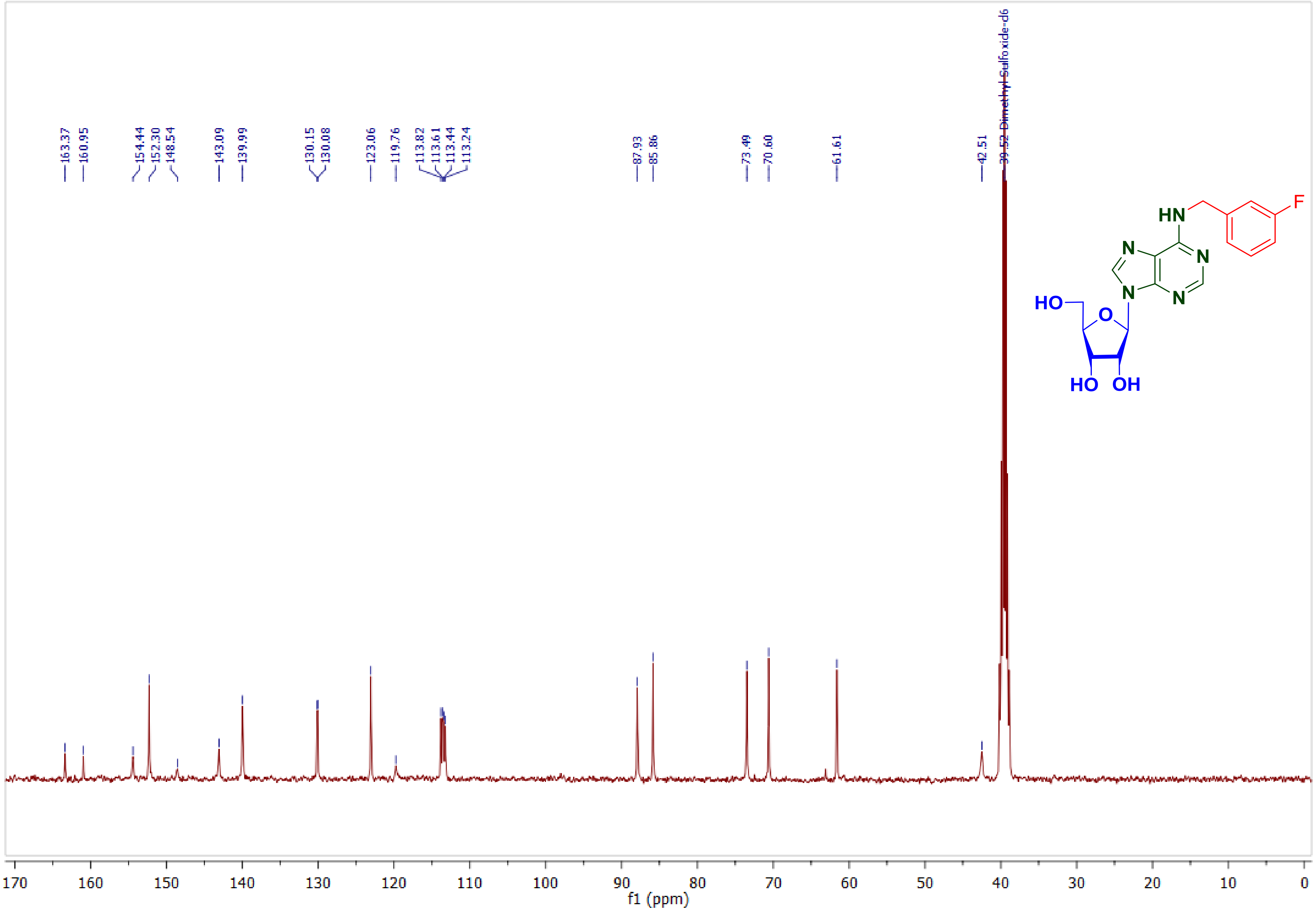

^{13}C-NMR-spectrum (100 MHz) of N^6-(3-fluorobenzyl)-adenosine (**6**) in DMSO-d_6 at 303 K

Acquisition Parameter

Source Type	ESI	Ion Polarity	Positive	Set Nebulizer	0.4 Bar
Focus	Active	Set Capillary	4500 V	Set Dry Heater	200 °C
Scan Begin	50 m/z	Set End Plate Offset	-500 V	Set Dry Gas	4.0 l/min
Scan End	3000 m/z	Set Charging Voltage	2000 V	Set Divert Valve	Source
		Set Corona	0 nA	Set APCI Heater	0 °C

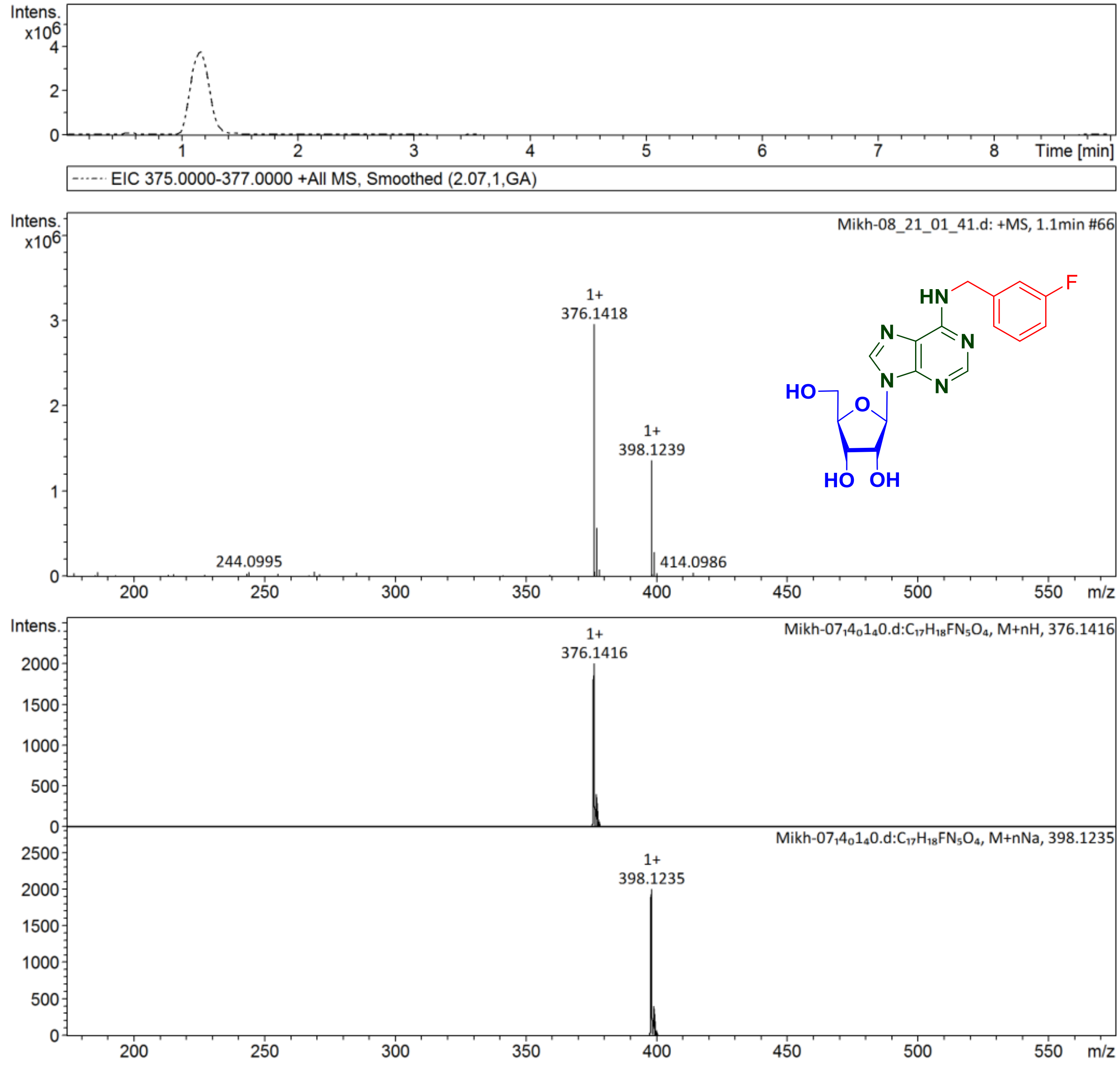

High-resolution mass spectrum (HRMS) of N^6-(3-fluorobenzyl)-adenosine (**6**)

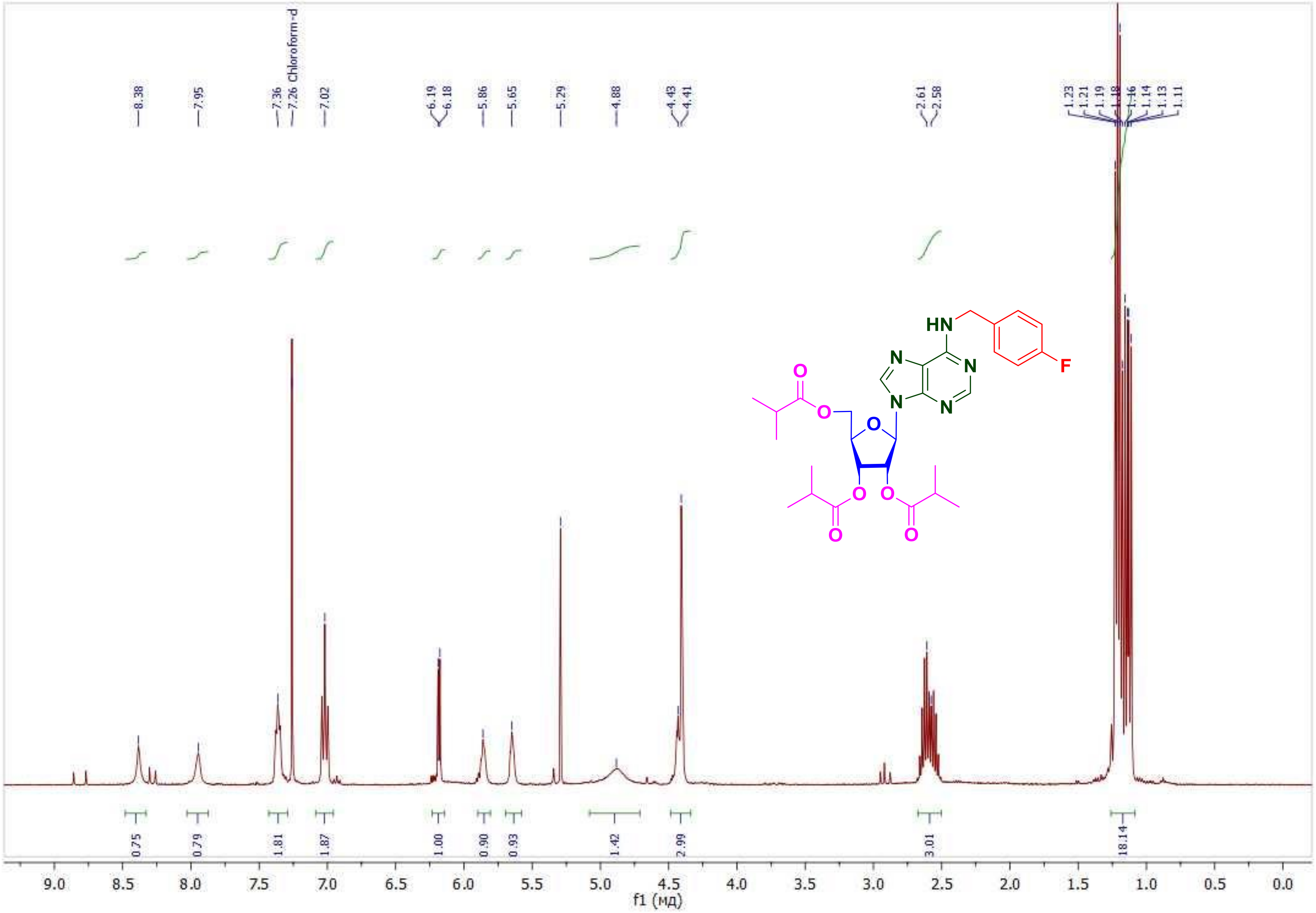

^{1}H-NMR-spectrum (400 MHz) of N^6-(4-fluorobenzyl)-2′,3′,5′-tri-O-isobutyroyladenosine in $CDCl_3$ at 303 K

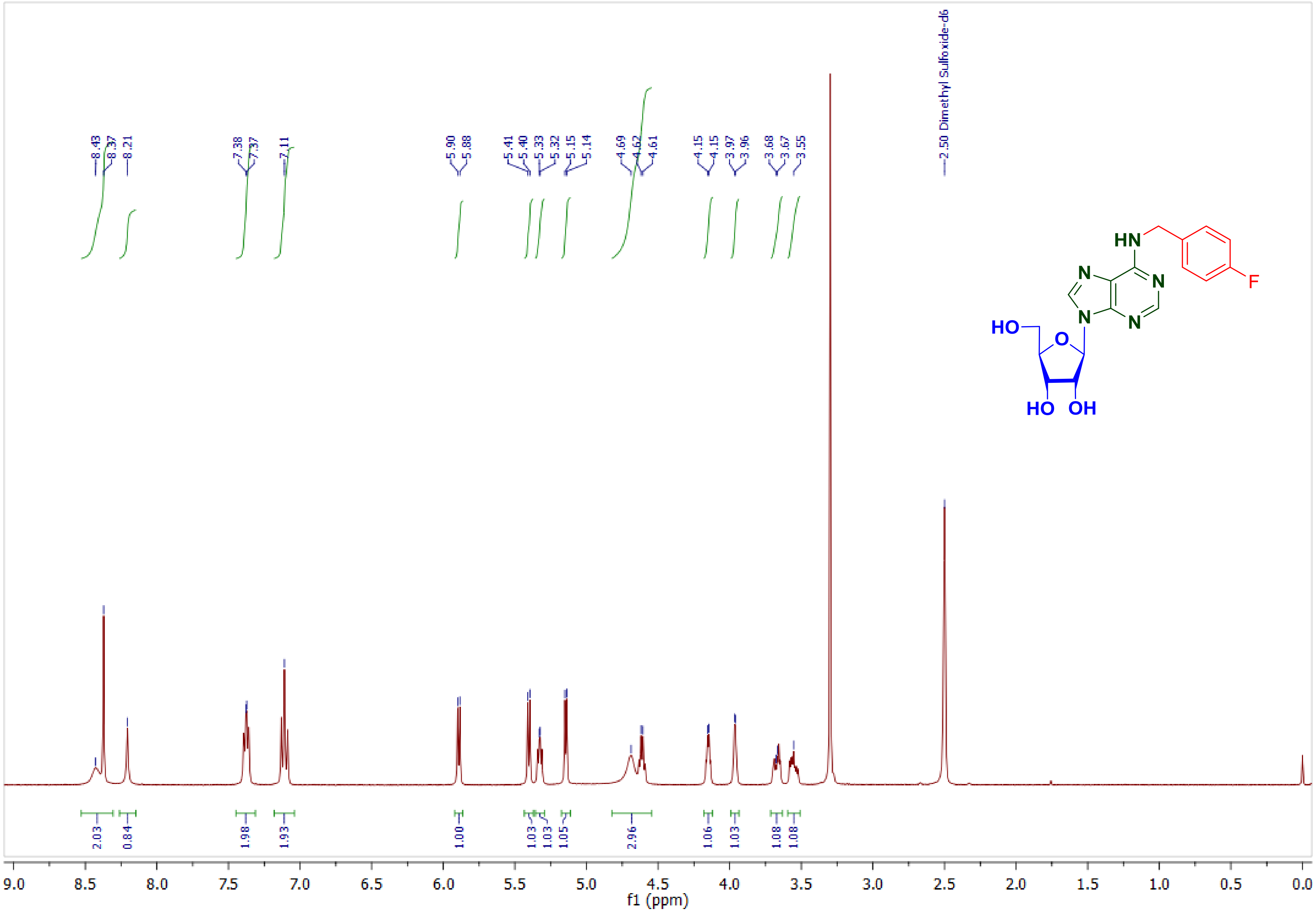

^{1}H-NMR-spectrum (400 MHz) of N^6-(4-fluorobenzyl)-adenosine (**7**) in DMSO-d_6 at 303 K

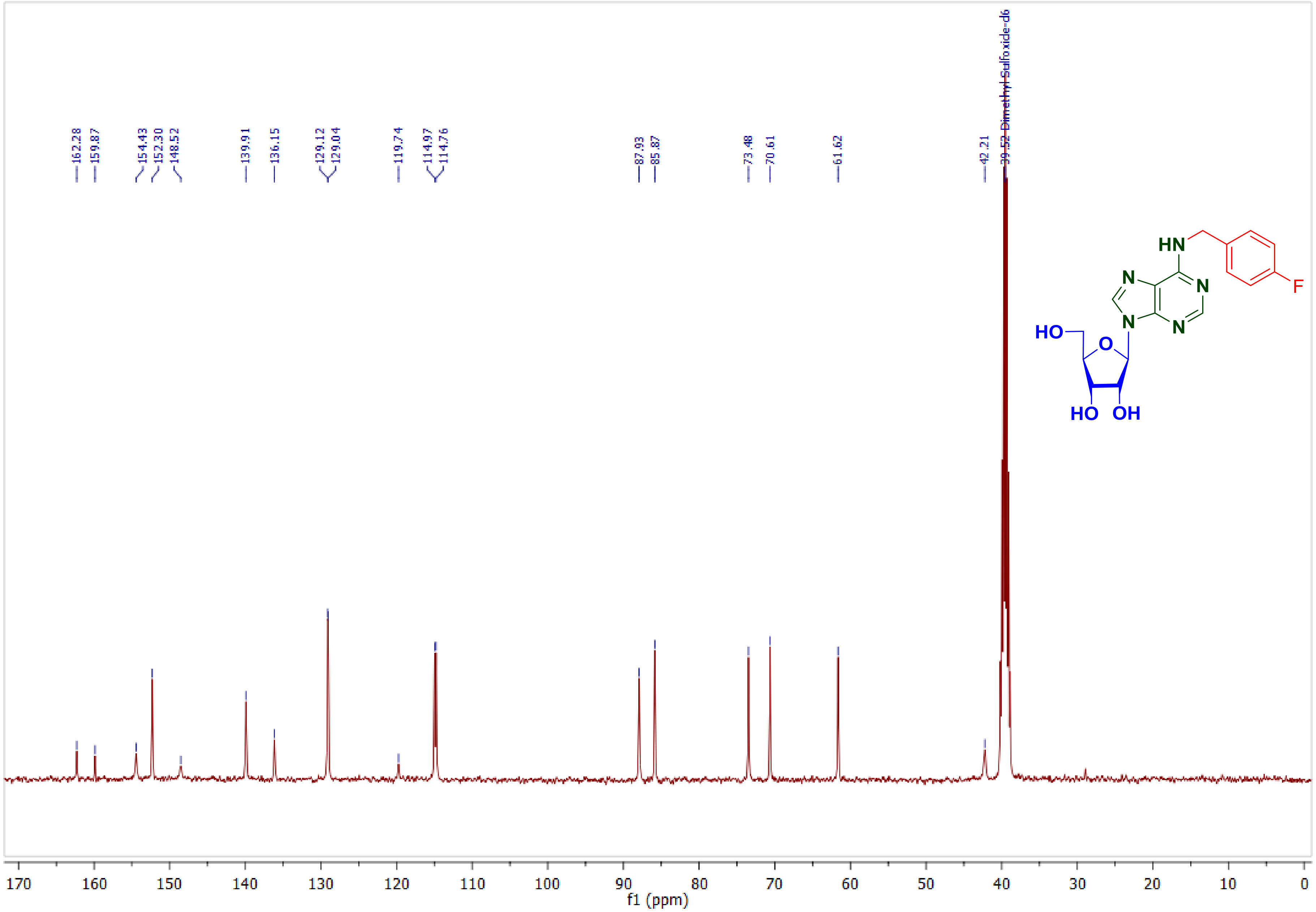

^{13}C-NMR-spectrum (100 MHz) of N^6-(4-fluorobenzyl)-adenosine (**7**) in DMSO-d_6 at 303 K

Acquisition Parameter

Source Type	ESI	Ion Polarity	Positive	Set Nebulizer	0.4 Bar
Focus	Active	Set Capillary	4500 V	Set Dry Heater	200 °C
Scan Begin	50 m/z	Set End Plate Offset	-500 V	Set Dry Gas	4.0 l/min
Scan End	3000 m/z	Set Charging Voltage	2000 V	Set Divert Valve	Source
		Set Corona	0 nA	Set APCI Heater	0 °C

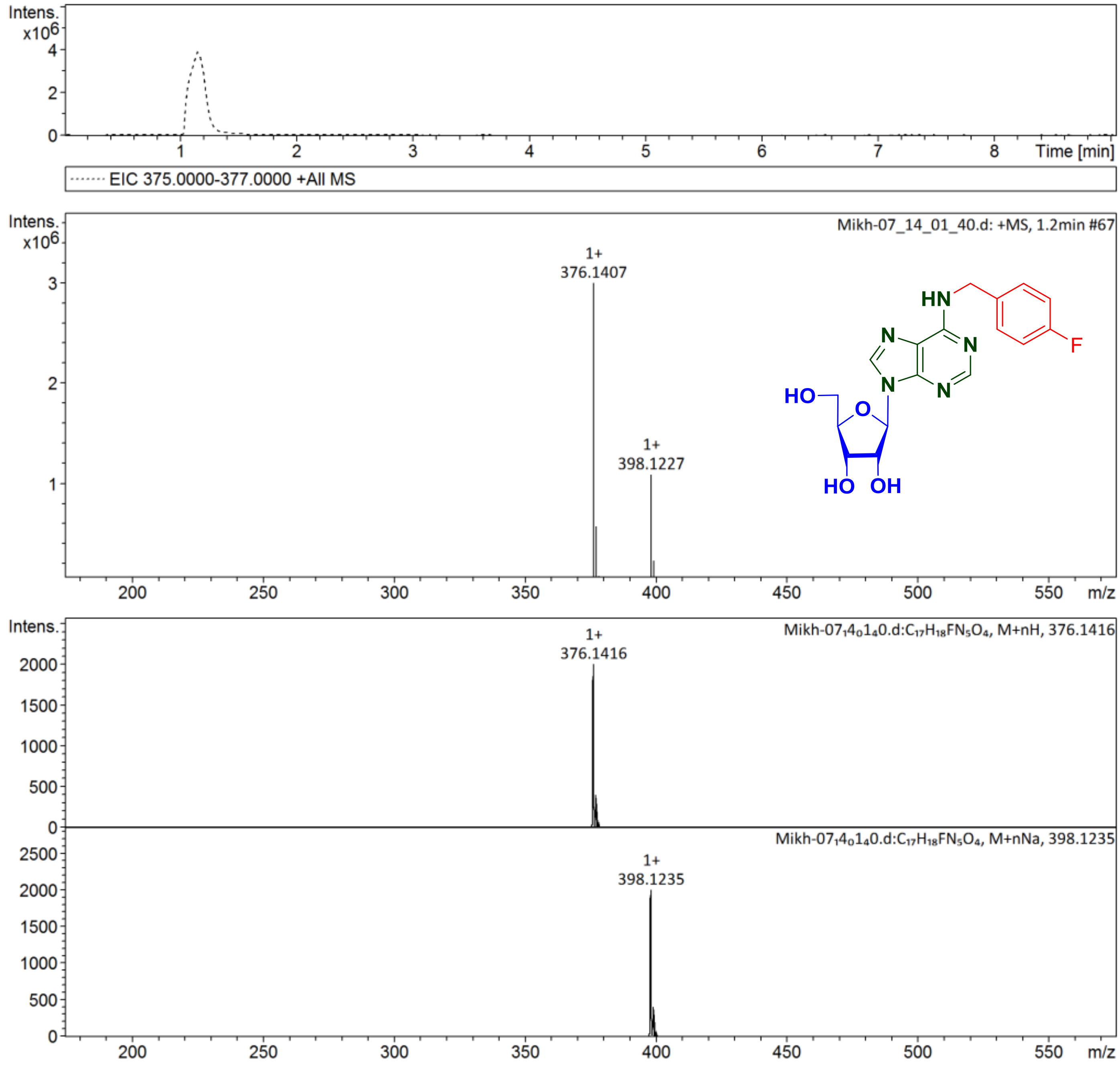

High-resolution mass spectrum (HRMS) of N^6-(4-fluorobenzyl)-adenosine (**7**)

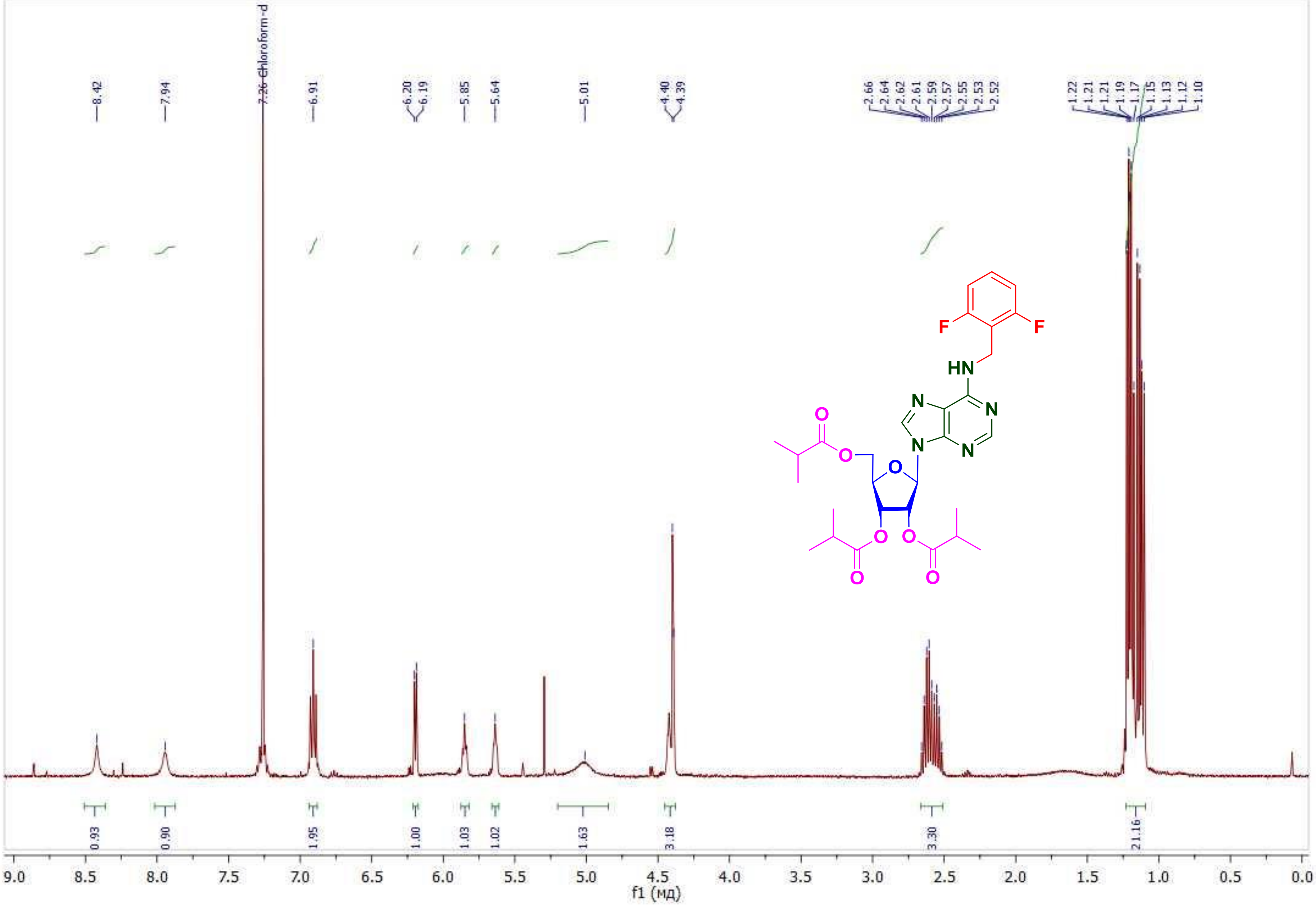

^{1}H-NMR-spectrum (400 MHz) of N^6-(2,6-difluorobenzyl)-2′,3′,5′-tri-O-isobutyroyladenosine in $CDCl_3$ at 303 K

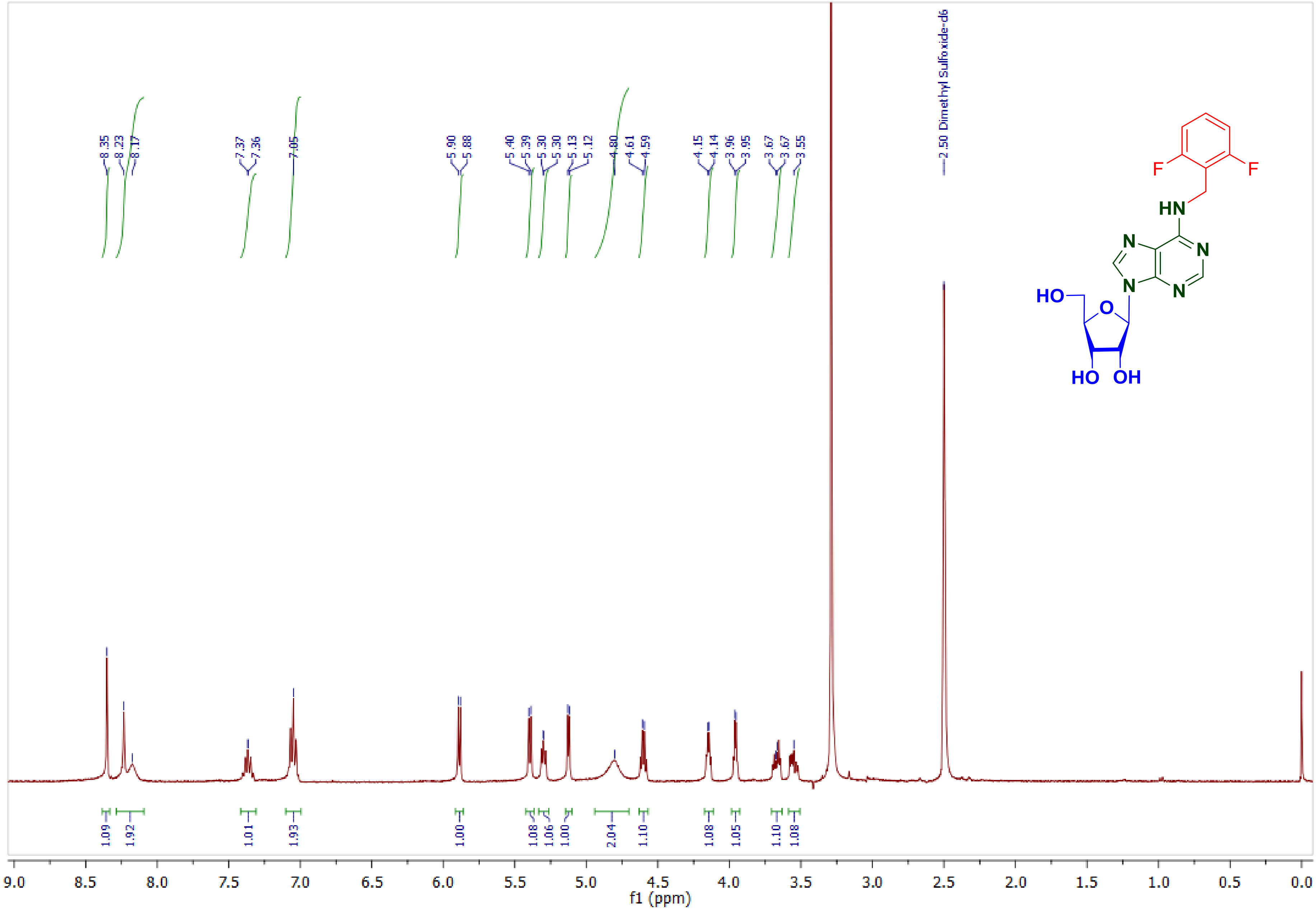

^{1}H-NMR-spectrum (400 MHz) of N^6-(2,6-difluorobenzyl)-adenosine (**8**) in DMSO-d_6 at 303 K

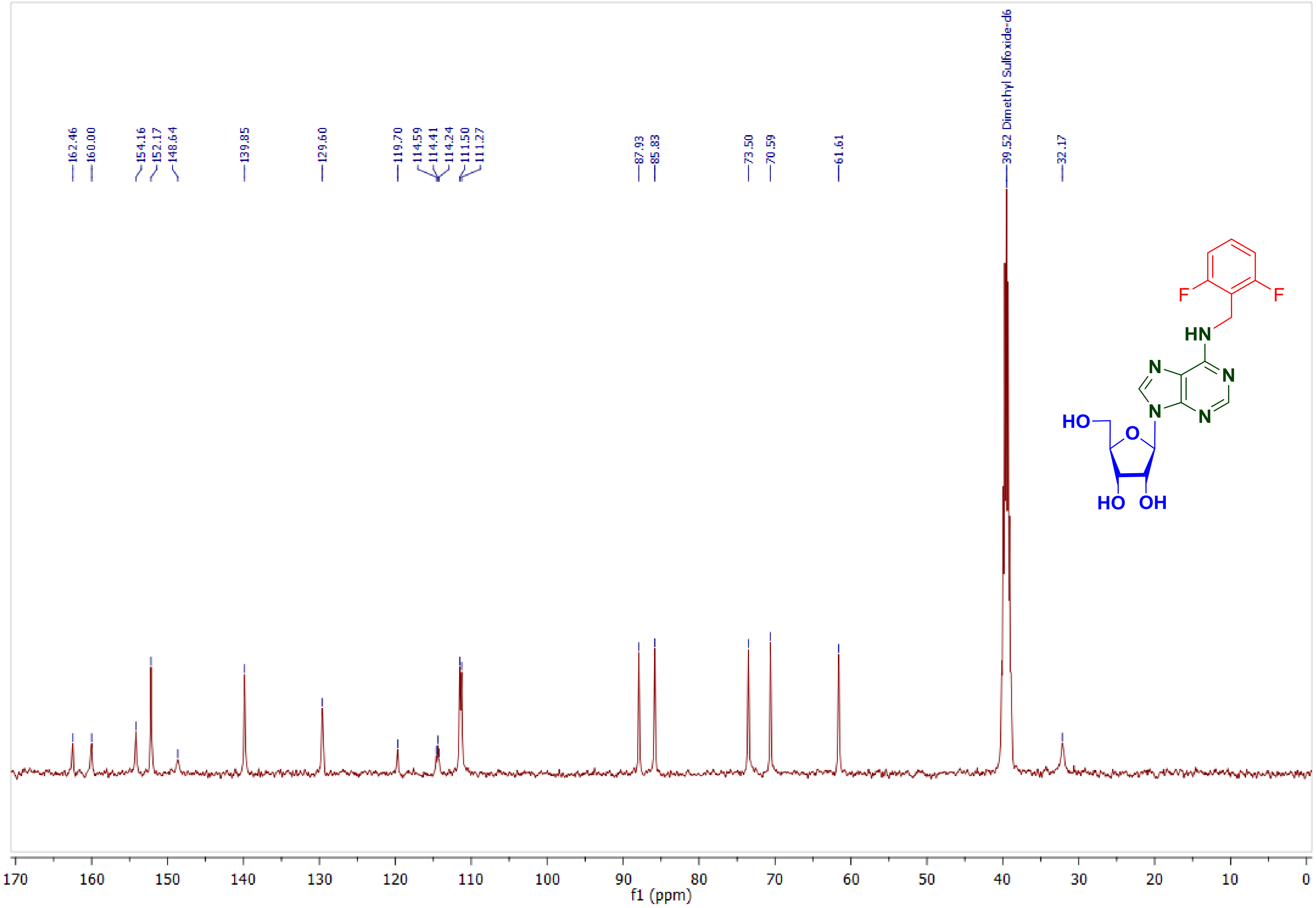

^{13}C-NMR-spectrum (100 MHz) of N^6-(2,6-difluorobenzyl)-adenosine (**8**) in DMSO-d_6 at 303 K

Acquisition Parameter

Source Type	ESI	Ion Polarity	Positive	Set Nebulizer	0.4 Bar
Focus	Active	Set Capillary	4500 V	Set Dry Heater	200 °C
Scan Begin	50 m/z	Set End Plate Offset	-500 V	Set Dry Gas	4.0 l/min
Scan End	3000 m/z	Set Charging Voltage	2000 V	Set Divert Valve	Source
		Set Corona	0 nA	Set APCI Heater	0 °C

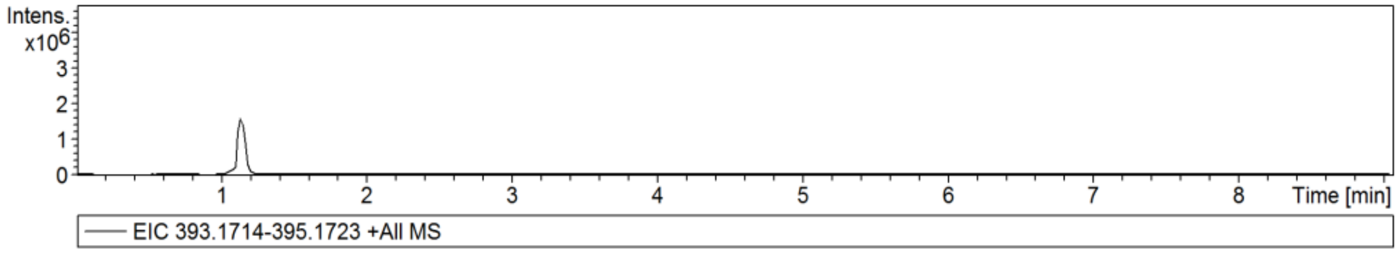

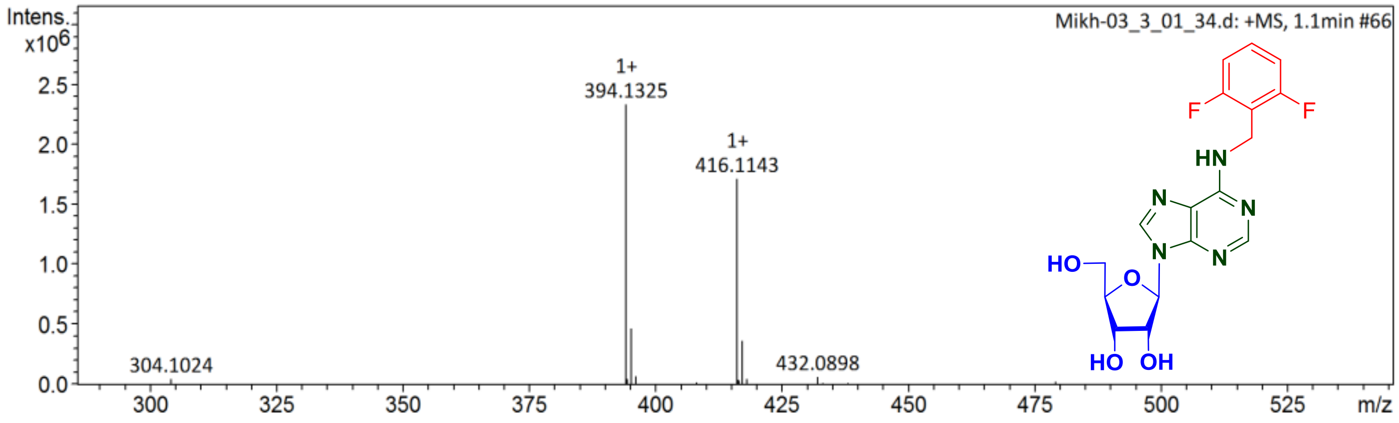

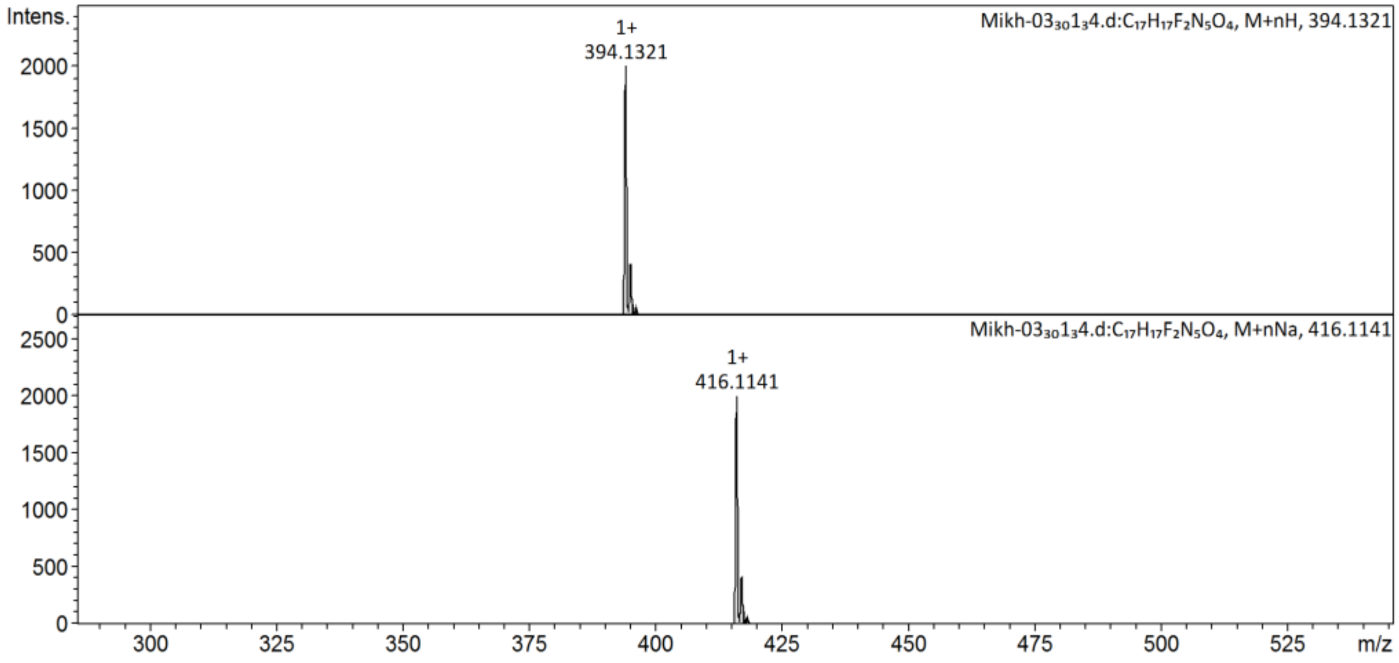

High-resolution mass spectrum (HRMS) of N^6-(2,6-difluorobenzyl)-adenosine (**8**)

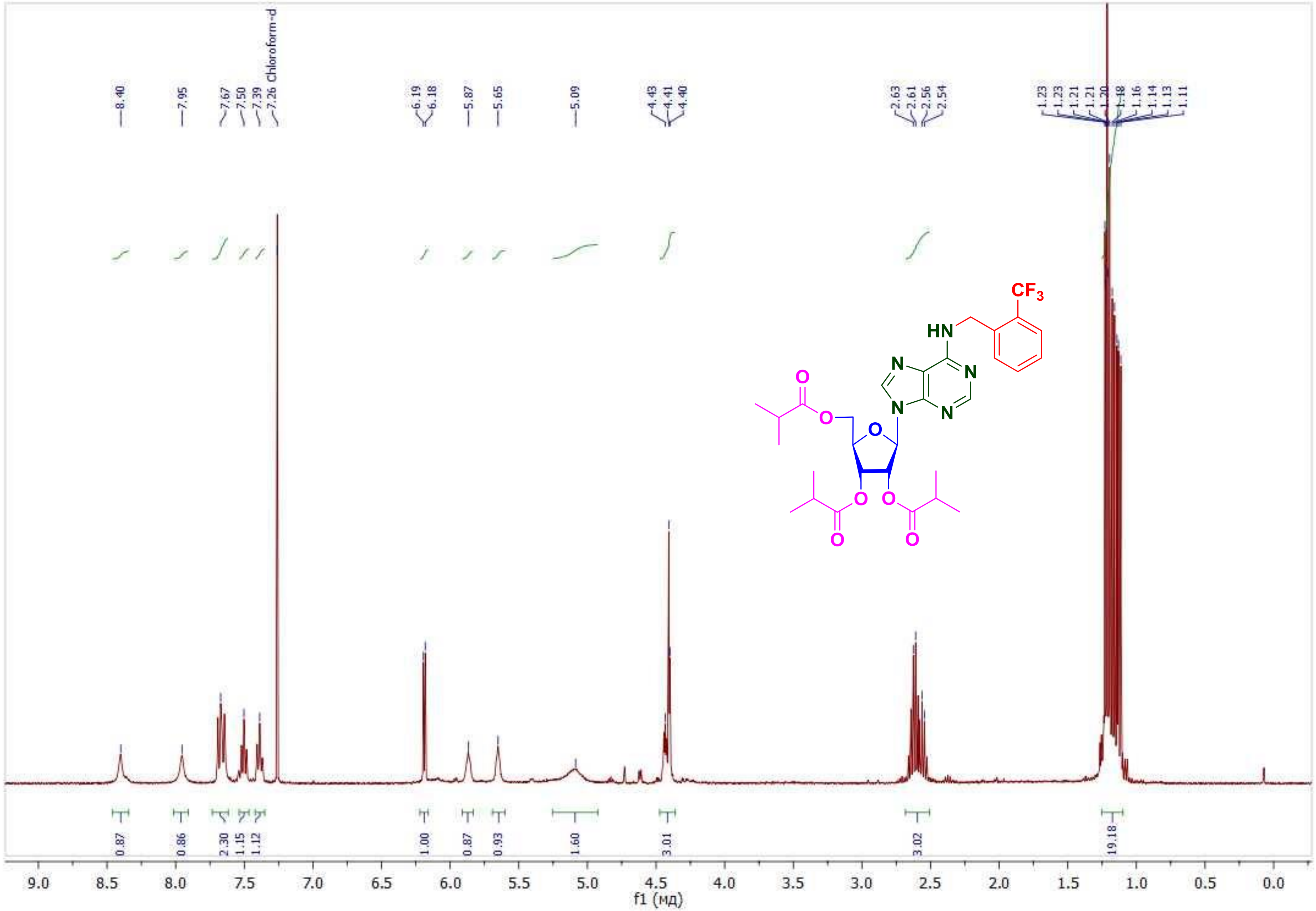

^{1}H-NMR-spectrum (400 MHz) of N^6-(2-trifluoromethylbenzyl)-2′,3′,5′-tri-O-isobutyroyladenosine in $CDCl_3$ at 303 K

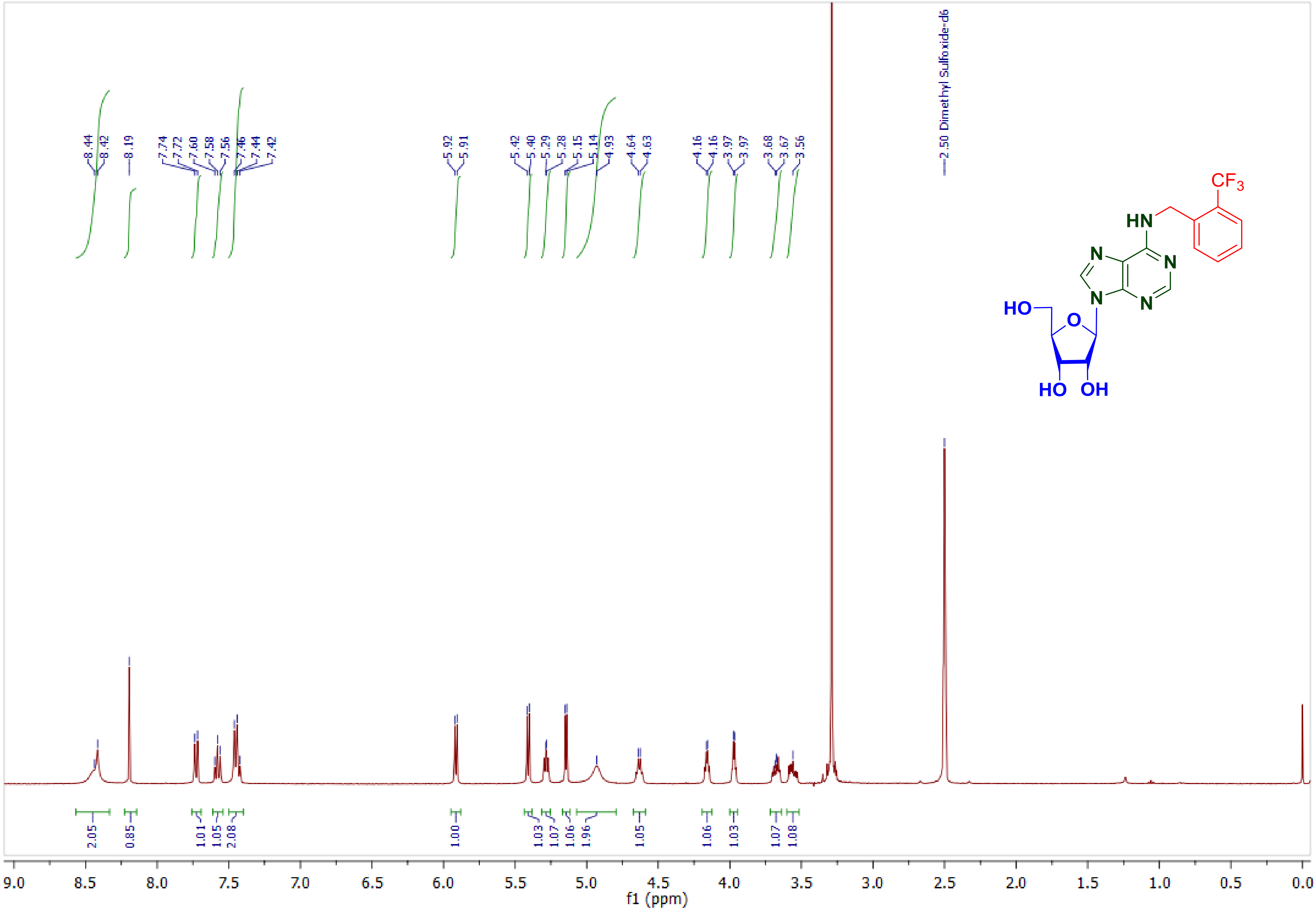

^{1}H-NMR-spectrum (400 MHz) of N^6-(2-trifluoromethylbenzyl)-adenosine (**9**) in DMSO-d_6 at 303 K

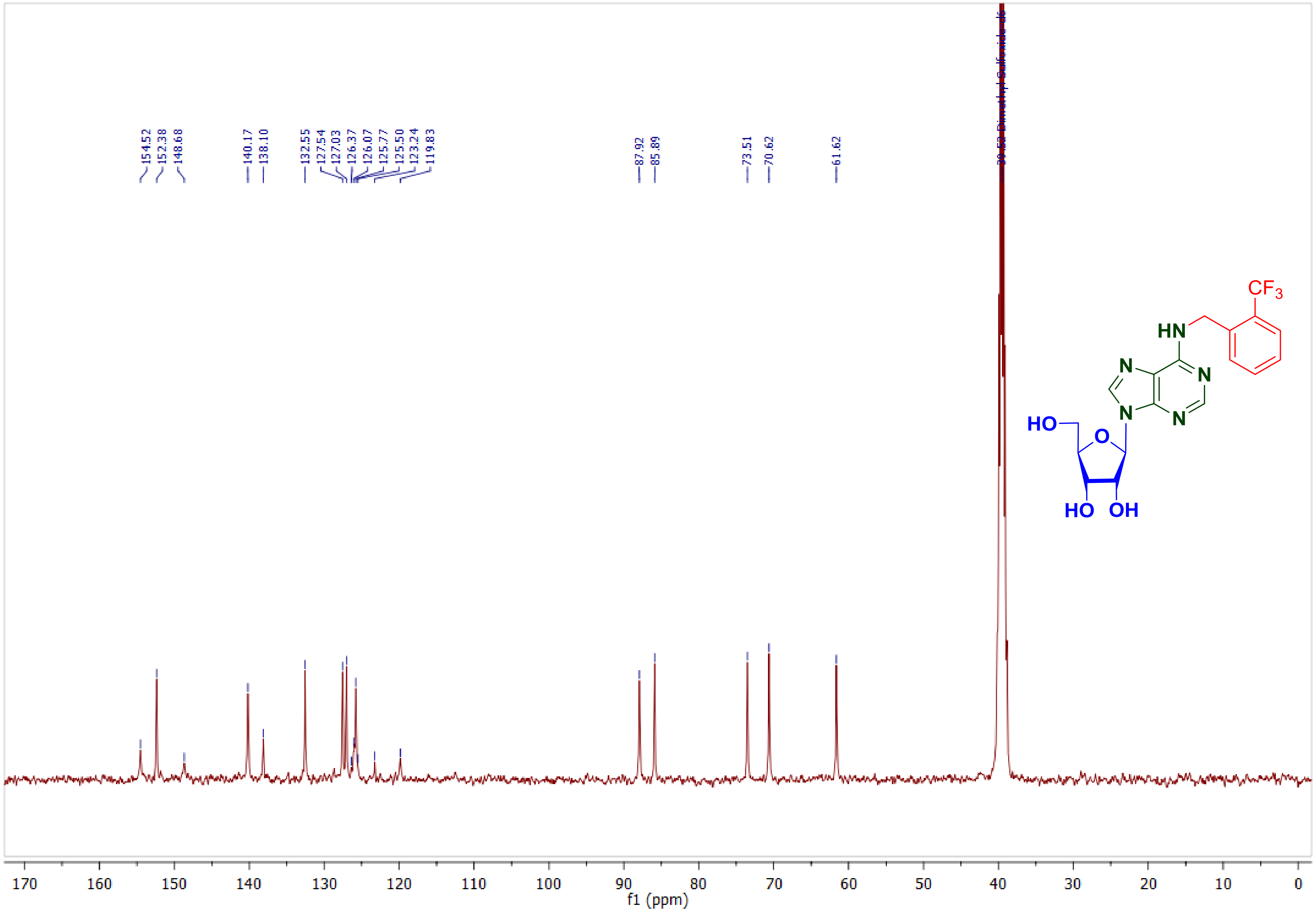

^{13}C-NMR-spectrum (100 MHz) of *N*^{6}-(2-trifluoromethylbenzyl)-adenosine (**9**) in DMSO-d_6 at 303 K

Acquisition Parameter

Source Type	ESI	Ion Polarity	Positive	Set Nebulizer	0.4 Bar
Focus	Active	Set Capillary	4500 V	Set Dry Heater	200 °C
Scan Begin	50 m/z	Set End Plate Offset	-500 V	Set Dry Gas	4.0 l/min
Scan End	3000 m/z	Set Charging Voltage	2000 V	Set Divert Valve	Source
		Set Corona	0 nA	Set APCI Heater	0 °C

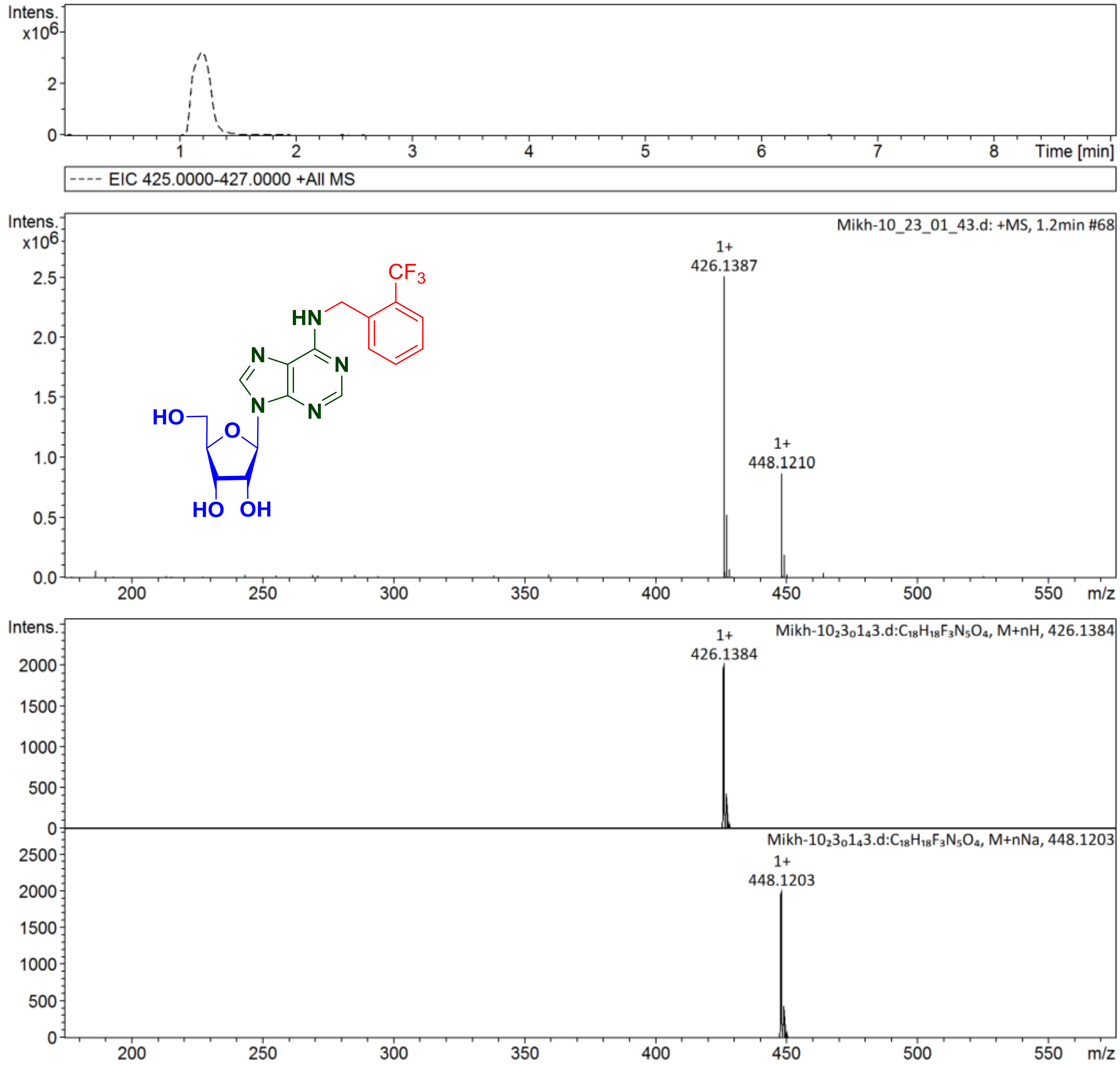

High-resolution mass spectrum (HRMS) of N^6-(2-trifluoromethylbenzyl)-adenosine (**9**)

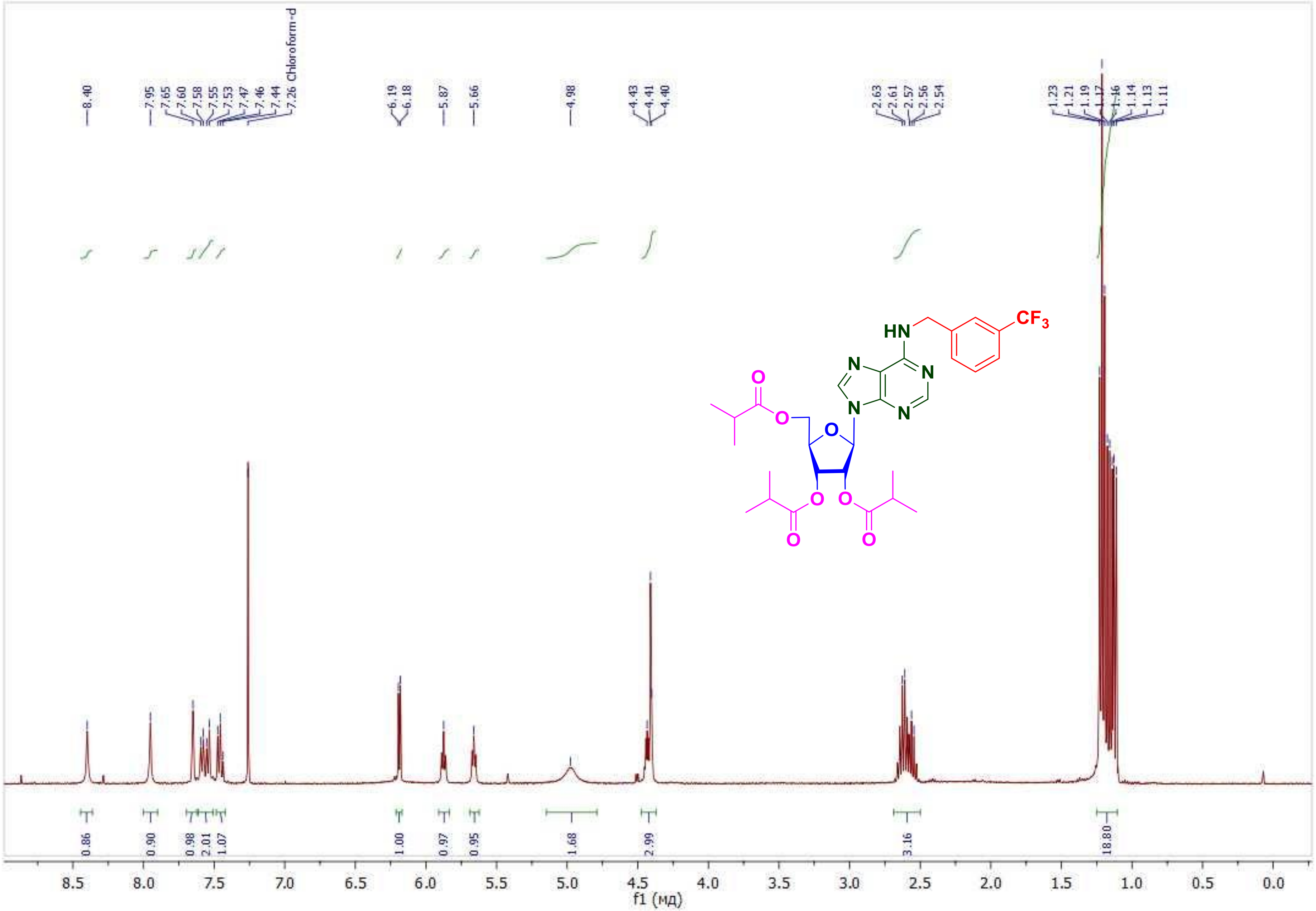

^{1}H-NMR-spectrum (400 MHz) of N^6-(3-trifluoromethylbenzyl)-2′,3′,5′-tri-O-isobutyroyladenosine in $CDCl_3$ at 303 K

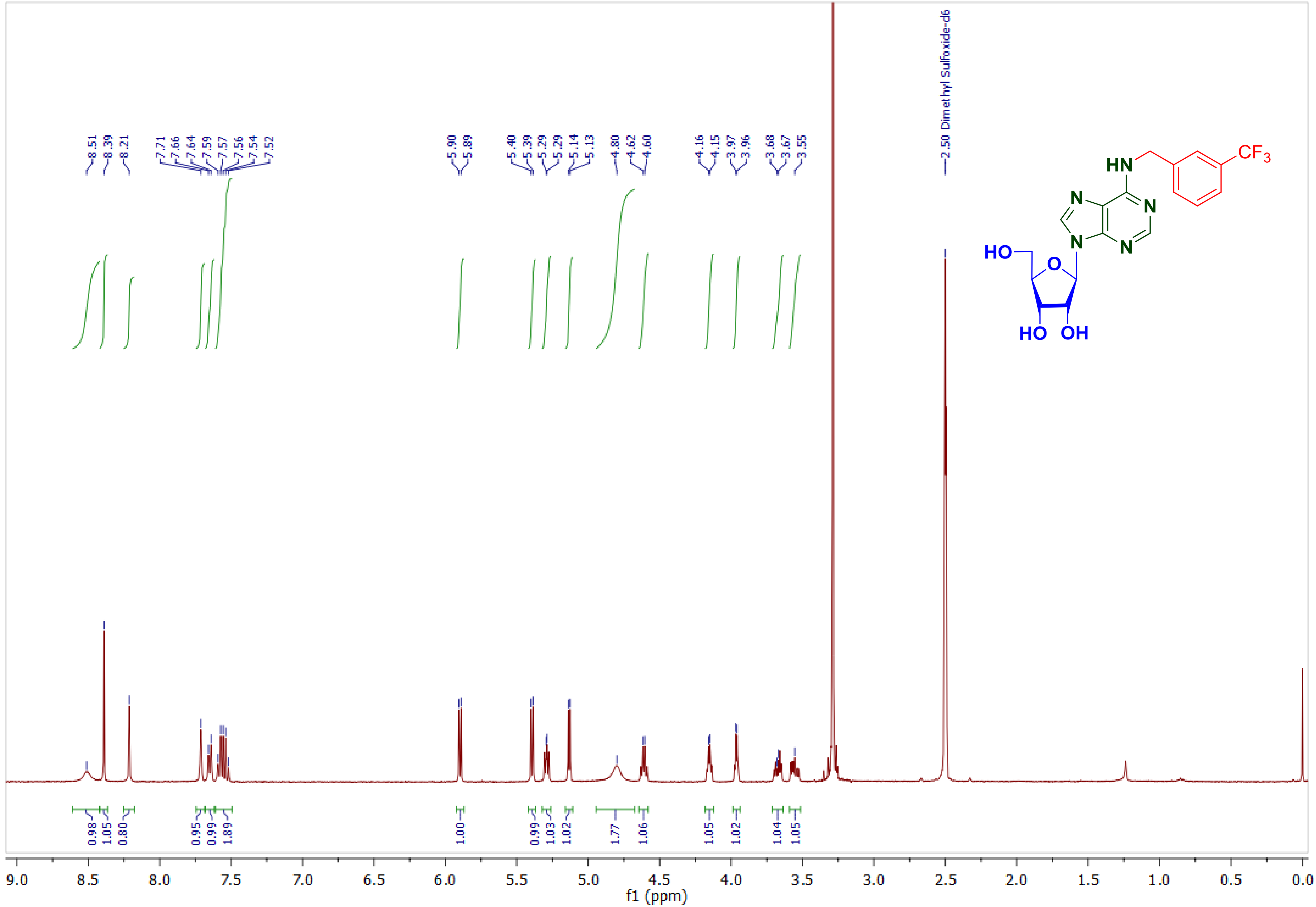

^{1}H-NMR-spectrum (400 MHz) of *N*[6]-(3-trifluoromethylbenzyl)-adenosine (**10**) in DMSO-d_6 at 303 K

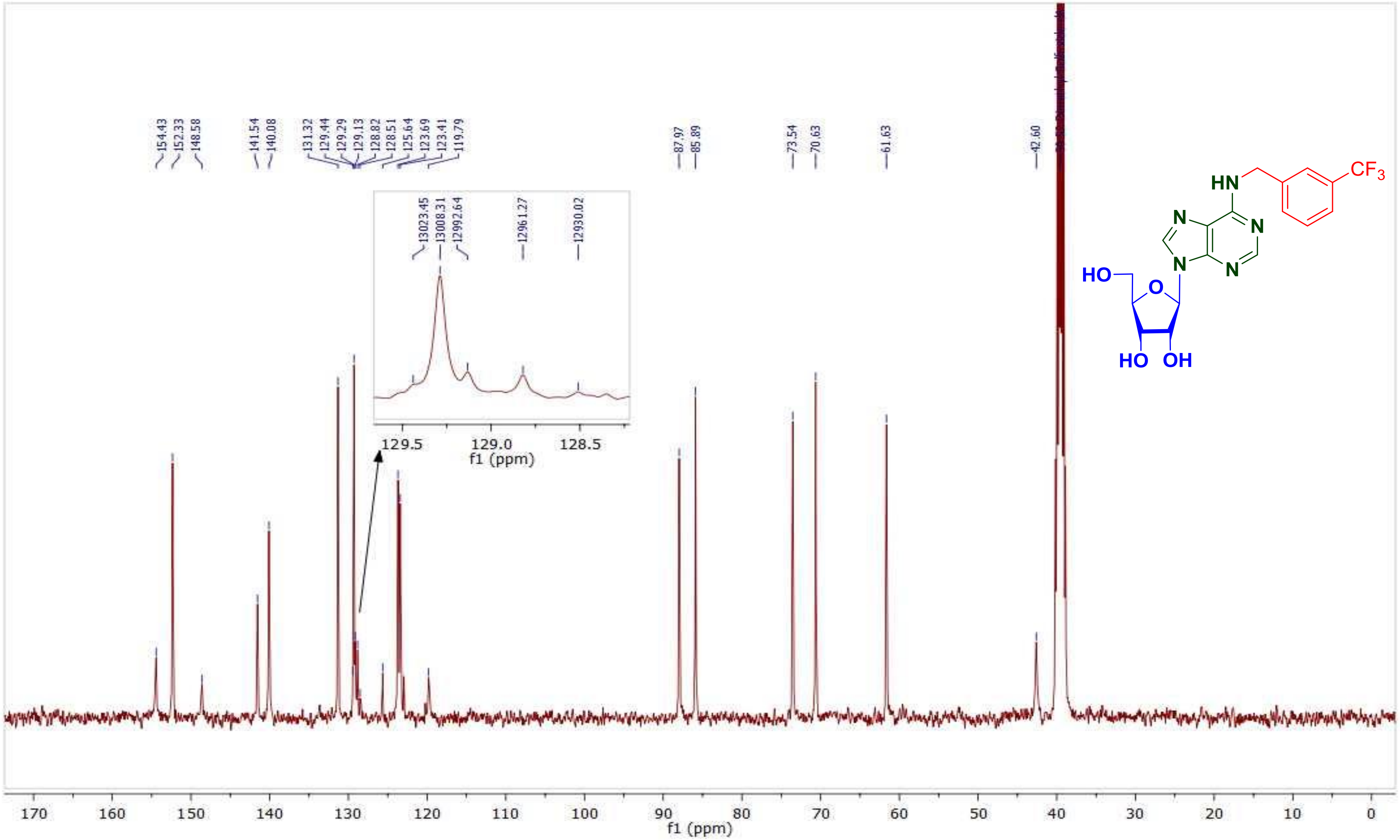

^{13}C-NMR-spectrum (100 MHz) of N^6-(3-trifluoromethylbenzyl)-adenosine (**10**) in DMSO-d_6 at 303 K

Acquisition Parameter

Source Type	ESI	Ion Polarity	Positive	Set Nebulizer	0.4 Bar
Focus	Active	Set Capillary	4500 V	Set Dry Heater	200 °C
Scan Begin	50 m/z	Set End Plate Offset	-500 V	Set Dry Gas	4.0 l/min
Scan End	3000 m/z	Set Charging Voltage	2000 V	Set Divert Valve	Source
		Set Corona	0 nA	Set APCI Heater	0 °C

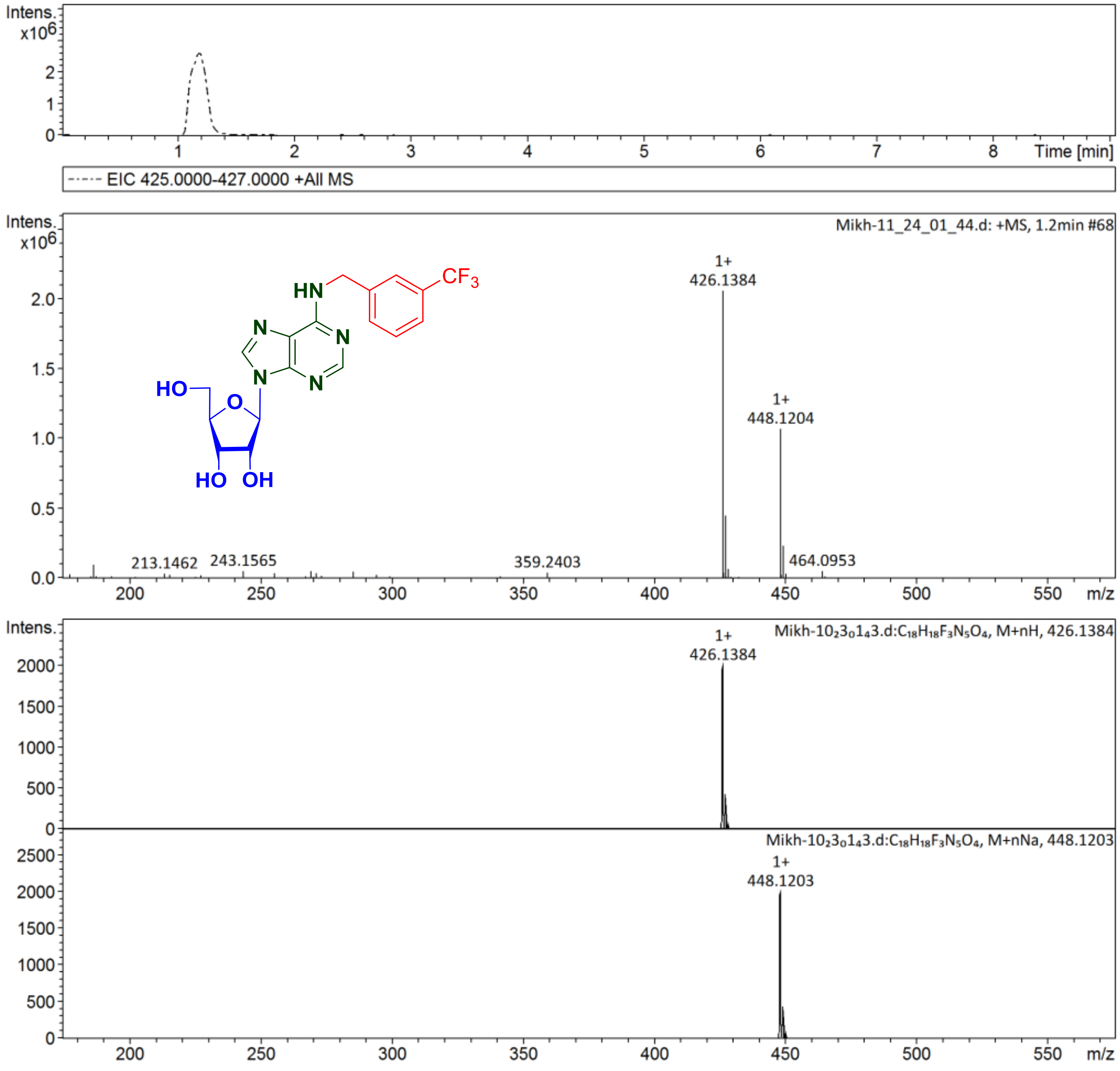

High-resolution mass spectrum (HRMS) of N^6-(3-trifluoromethylbenzyl)-adenosine (**10**)

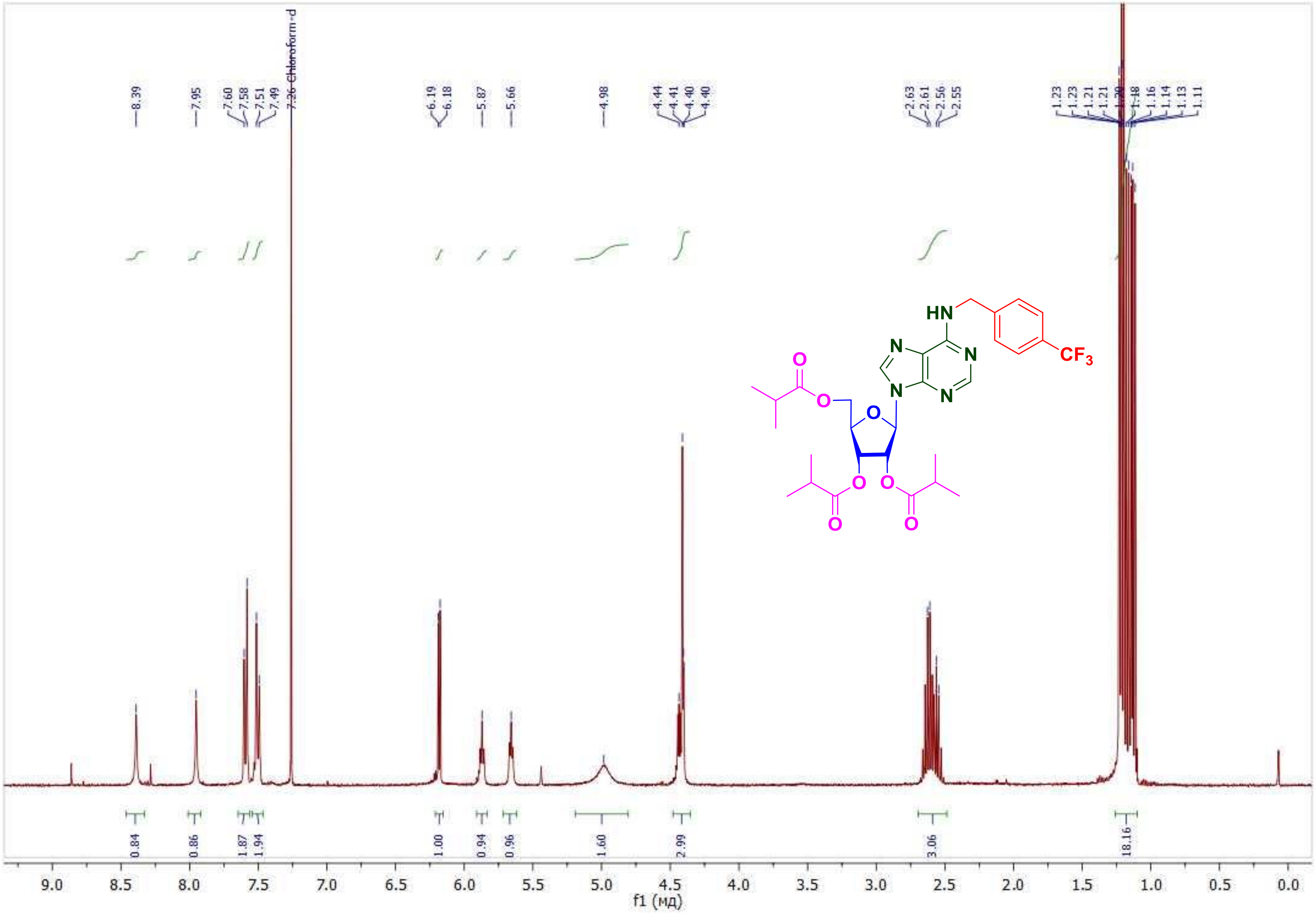

^{1}H-NMR-spectrum (400 MHz) of *N*^{6}-(4-trifluoromethylbenzyl)-2′,3′,5′-tri-O-isobutyroyladenosine in $CDCl_3$ at 303 K

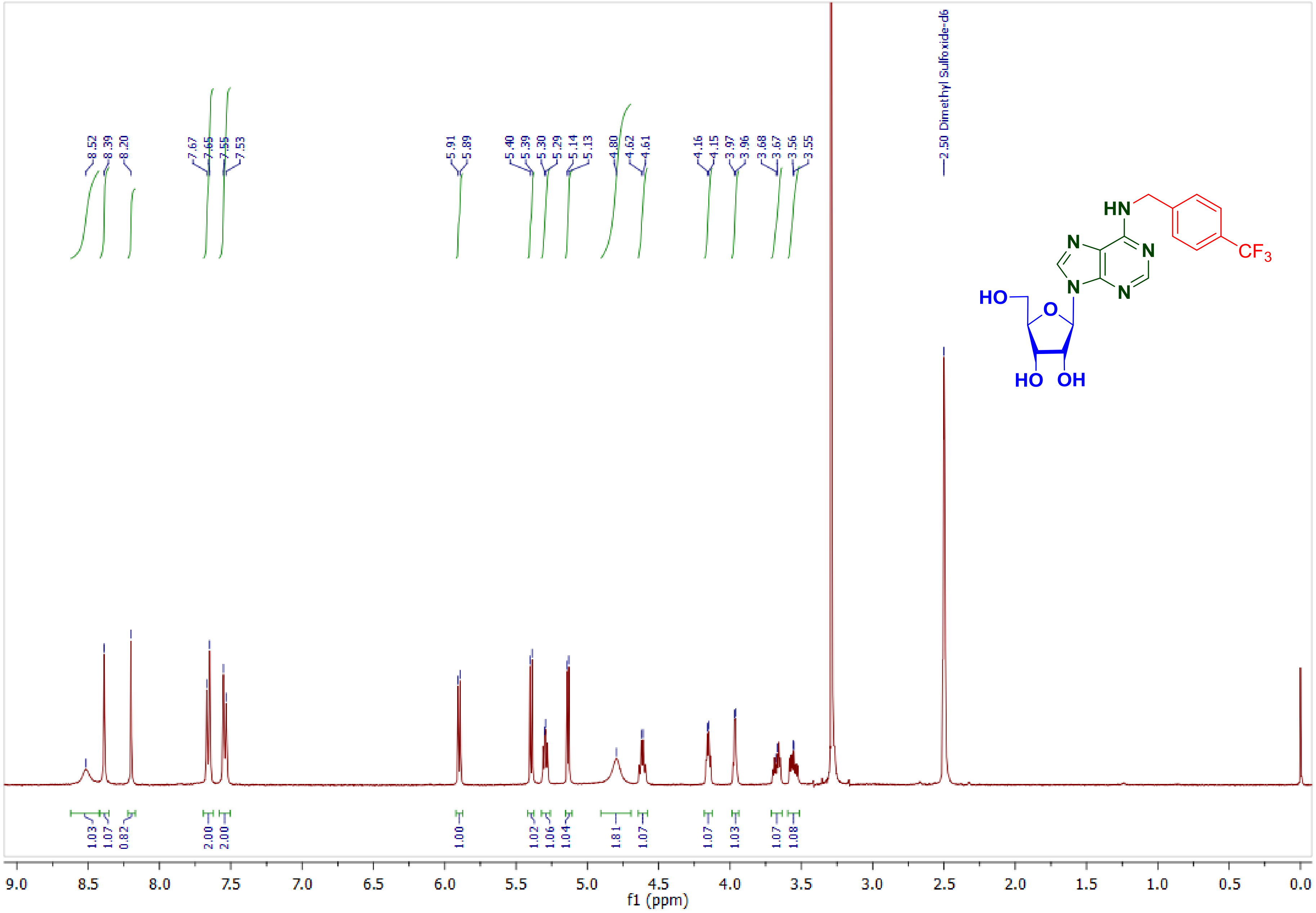

^{1}H-NMR-spectrum (400 MHz) of N^6-(4-trifluoromethylbenzyl)-adenosine (**11**) in DMSO-d_6 at 303 K

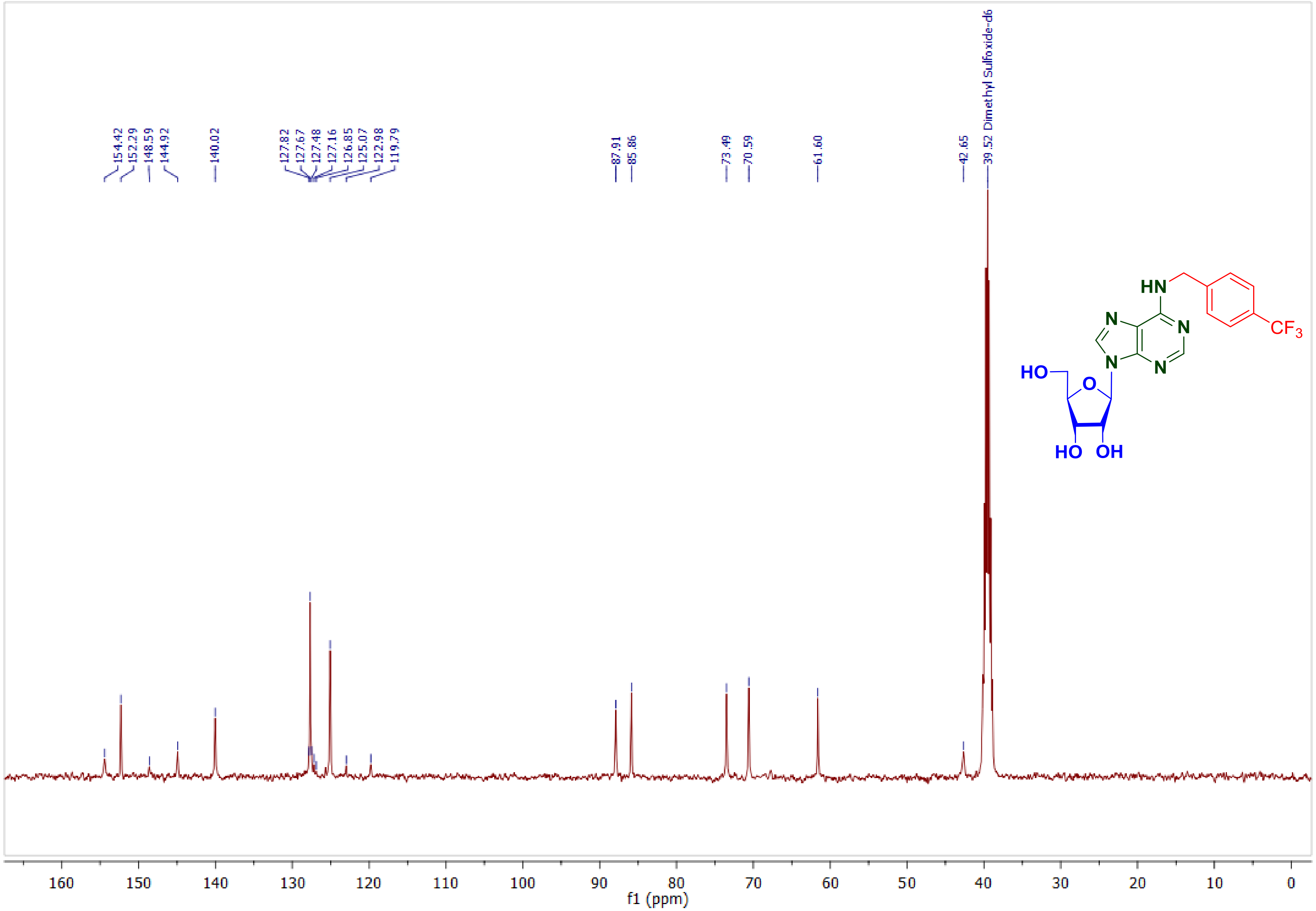

^{13}C-NMR-spectrum (100 MHz) of N^6-(4-trifluoromethylbenzyl)-adenosine (**11**) in DMSO-d_6 at 303 K

Acquisition Parameter

Source Type	ESI	Ion Polarity	Positive	Set Nebulizer	0.4 Bar
Focus	Active	Set Capillary	4500 V	Set Dry Heater	200 °C
Scan Begin	50 m/z	Set End Plate Offset	-500 V	Set Dry Gas	4.0 l/min
Scan End	3000 m/z	Set Charging Voltage	2000 V	Set Divert Valve	Source
		Set Corona	0 nA	Set APCI Heater	0 °C

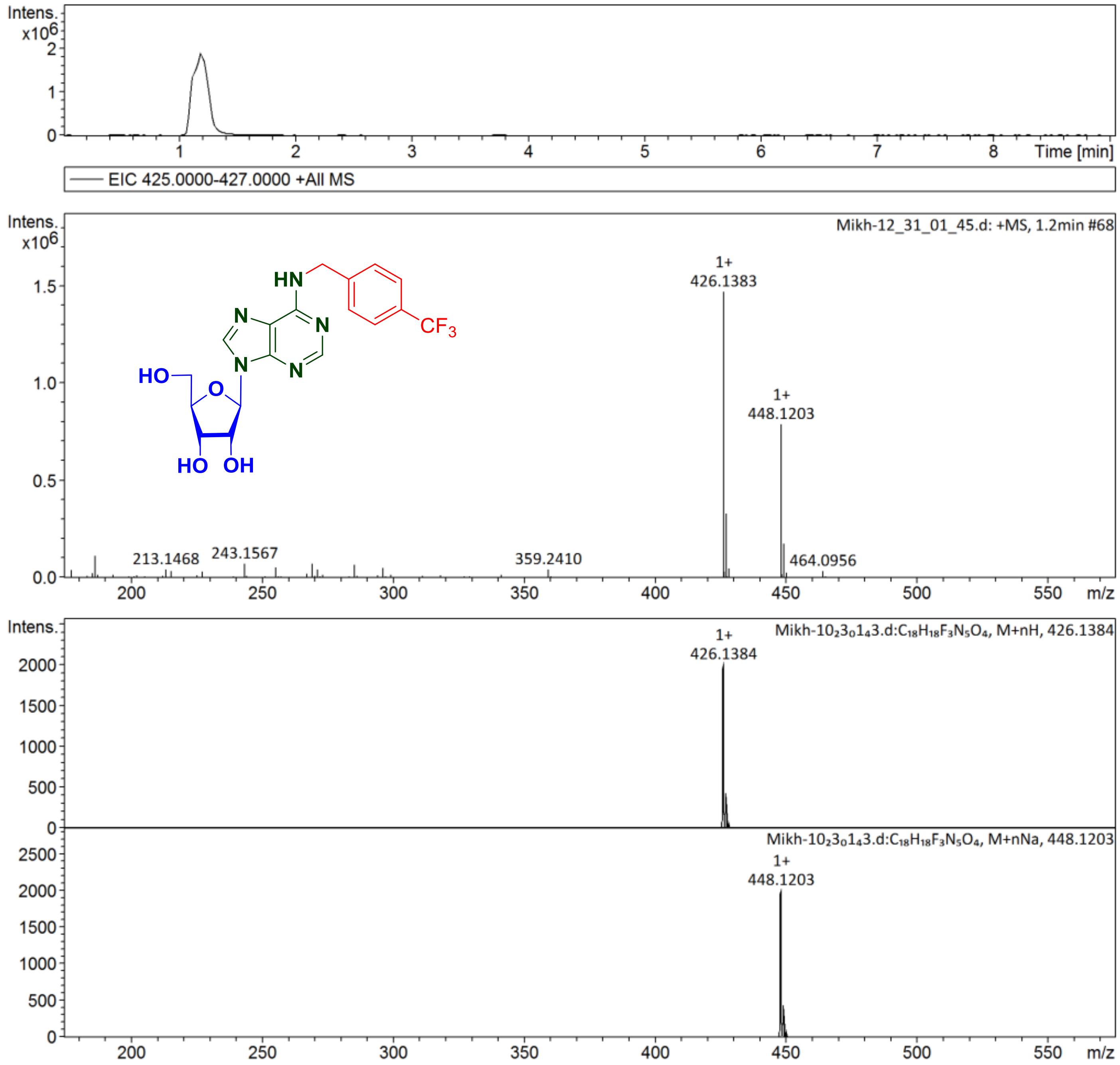

High-resolution mass spectrum (HRMS) of N^6-(4-trifluoromethylbenzyl)-adenosine (**11**)
